# Protection of Tregs, Suppression of Th1 and Th17 Cells, and Amelioration of Experimental Allergic Encephalomyelitis by a Physically-Modified Saline

**DOI:** 10.1371/journal.pone.0051869

**Published:** 2012-12-20

**Authors:** Susanta Mondal, Jeffrey A. Martinson, Supurna Ghosh, Richard Watson, Kalipada Pahan

**Affiliations:** 1 Department of Neurological Sciences, Rush University Medical Center, Chicago, Illinois, United States of America; 2 Department of Immunology and Microbiology, Rush University Medical Center, Chicago, Illinois, United States of America; 3 Revalesio Corporation, Tacoma, Washington, United States of America; CNRS, France

## Abstract

In multiple sclerosis (MS) and other autoimmune diseases, the autoreactive T cells overcome the resistance provided by the regulatory T cells (Tregs) due to a decrease in the number of Foxp3-expressing Tregs. Therefore, upregulation and/or maintenance of Tregs during an autoimmune insult may have therapeutic efficacy in autoimmune diseases. Although several immunomodulatory drugs and molecules are available, most present significant side effects over long-term use. Here we have undertaken an innovative approach to upregulate Tregs and achieve immunomodulation. RNS60 is a 0.9% saline solution generated by subjecting normal saline to Taylor-Couette-Poiseuille (TCP) flow under elevated oxygen pressure. RNS60, but not NS (normal saline), RNS10.3 (TCP-modified saline without excess oxygen) and PNS60 (saline containing excess oxygen without TCP modification), was found to upregulate Foxp3 and enrich Tregs in MBP-primed T cells. Moreover, RNS60, but not NS, RNS10.3 and PNS60, inhibited the production of nitric oxide (NO) and the expression of iNOS in MBP-primed splenocytes. Incubation of the cells with an NO donor abrogated the RNS60-mediated upregulation of Foxp3. These results suggest that RNS60 boosts Tregs via suppression of NO production. Consistent to the suppressive activity of Tregs towards autoreactive T cells, RNS60, but not NS, RNS10.3, or PNS60, suppressed the differentiation of Th17 and Th1 cells and shifted the balance towards a Th2 response. Finally, RNS60 treatment exhibited immunomodulation and ameliorated adoptive transfer of experimental allergic encephalomyelitis, an animal model of MS, via Tregs. These results describe a novel immunomodulatory property of RNS60 and suggest its exploration for therapeutic intervention in MS and other autoimmune disorders.

## Introduction

Regulatory T cells (Tregs), a special subset of T cells, serve as a primary regulator for the immune response that maintains homeostasis between immune activation and immune suppression [1], [2]. A misguided and over active immune response against self and non-self antigens is physiologically harmful and may underlie the development of various chronic inflammatory and autoimmune diseases. Tregs suppress activation and proliferation of self-reactive T cells and thereby inhibit immune response of self-reactive T cells against self-antigens [1], [2]. There are several kinds of Tregs, including naturally occurring, inducible, and IL-10–producing Tregs, and several controversies lie in choosing proper parameters that specifically characterize a particular kind of Treg [2], [3]. Irrespective of these discrepancies, recent advancements in research have established the transcription factor forkhead box p3 (Foxp3) as the most specific marker of Tregs [1]. Foxp3^+^ CD4^+^CD25^+^ T cells are considered as the most common phenotype of Tregs [1], [4]. Under normal physiological conditions, Tregs are able to suppress self-reactive T cells. However, during autoimmune pathogenesis, the immune system is dysregulated, resulting in a substantial decrease in the activity and the number of Tregs, and thereby leading to proliferation of self-reactive T cells and subsequent autoimmune attack.

The importance of Tregs in multiple sclerosis (MS) and experimental autoimmune encephalomyelitis (EAE), the animal model of MS, is becoming increasingly recognized. MS is associated with deficiency of Treg numbers and function [5], [6]. It has been shown that Tregs play a critical role in protection and recovery from EAE [7]. Although the exact mechanism of protection by Tregs is not clearly understood, it is suspected that Tregs exert protection by increasing the Th2 phenotype and decreasing the homing of autoreactive T cells [7]. Depletion of CD4^+^CD25^+^ cells inhibits natural recovery from EAE, whereas transfer of these cells to recipient mice reduces disease severity [8]. These observations imply that regulation of Tregs might play a decisive role in susceptibility to EAE. Recent studies suggest that the expression of Foxp3 and the numbers of peripheral CD4^+^CD25^+^ Foxp3^+^ T cells are significantly reduced in relapsing-remitting MS patients compared with those in control subjects [9]. Therefore, increasing and/or maintaining Tregs may be beneficial for treating MS.

Although there are other immunomodulatory compounds [10], [11], here we have tested a novel approach to achieve immunomodulation. RNS60 is a physically modified saline that contains no active pharmaceutical ingredients. RNS60 is generated by subjecting normal saline to Taylor-Couette-Poiseuille flow under elevated oxygen pressure [12]. Here we delineate that RNS60 increased the expression of Foxp3 and enriched T cell populations for Tregs via decreasing the level of nitric oxide. Accordingly, RNS60 suppressed Th1 and Th17 responses and augmented Th2 response. Finally in the animal model, RNS60 treatment was capable of increasing the proportion of Tregs and Th2, and suppressing the relative abundance of Th1 and Th17 cells, thus ameliorating the disease process of relapsing-remitting EAE. Furthermore, abrogation of the RNS60-mediated protection from EAE by anti-CD25 antibody suggests that the protective effect of RNS60 is mediated via Tregs. Our studies suggest that this physically-modified saline may be used to control aberrant immune responses in MS and other autoimmune pathologies.

## Materials and Methods

Animal maintaining and experiments were in accordance with National Institute of Health guidelines and were approved by the Institutional Animal Care and Use committee (IACUC#09-072) of the Rush University of Medical Center, Chicago, IL. Animals exhibiting paralysis were kept on soft bed and fed and watered through animal feeding needles. However, if any mouse came to the moribund stage, it was decapitated after anesthesia with ketamine/xylazine injectables.

### Reagents

Bovine myelin basic protein (MBP), L-glutamine and β-mercaptoethanol were obtained from Invitrogen (Carlsbad, CA). Fetal bovine serum (FBS) and RPMI 1640 were from Mediatech (Washington, DC). Heat-killed *M. tuberculosis* (H37RA) was purchased from Difco Labs. Incomplete Freund's adjuvant (IFA) was obtained from Calbiochem. Solvent Blue 38, cresyl violet acetate, and lithium carbonate were purchased from Sigma (St. Louis, MO).

### Induction of EAE

Specific pathogen-free female SJL/J mice (3–4 weeks old) were purchased from Harlan Sprague-Dawley (Indianapolis, IN). Donor mice were immunized s.c. with 400 µg bovine MBP and 60 µg *M. tuberculosis* in IFA [13], [14], [15], [16]. Animals were killed 10–12 days postimmunization, and the draining lymph nodes were harvested. Single cell suspensions were treated with RBC lysis buffer (Sigma-Aldrich), washed, and cultured at a concentration of 4–5×10^6^ cells/mL in six-well plates in RPMI 1640 supplemented with 10% FBS, 50 µg/mL MBP, 50 µM 2-ME, 2 mM L-glutamine, 100 U/mL penicillin, and 100 µg/ml streptomycin. On day 4, cells were harvested and resuspended in HBSS. A total of 2×10^7^ viable cells in a volume of 200 µL were injected into the tail vein of naive mice. Pertussis toxin (150 ng/mouse; Sigma-Aldrich) was injected once via i.p. route on 0 day posttransfer (dpt) of cells. Cells isolated from donor mice immunized with CFA or IFA alone were not viable after 4 days in culture with MBP, and therefore were not transferred. Animals were observed daily for clinical symptoms. Experimental animals were scored by a masked investigator, as follows: 0, no clinical disease; 0.5, piloerection; 1, tail weakness; 1.5, tail paralysis; 2, hind limb weakness; 3, hind limb paralysis; 3.5, forelimb weakness; 4, forelimb paralysis; 5, moribund or death.

### Isolation of MBP-primed LNC and splenocytes

Lymph node cells (LNC) and splenocytes isolated from MBP-immunized mice as described above were cultured at a concentration of 2.0×10^6^ cells per ml in 12-well plates in the presence of 50 µg/ml MBP with or without different treatments for 24, 48 or 96 h. The nonadherent LNC or splenic T cells were collected and used for RNA isolation and FACS analysis.

### Preparation of RNS60

RNS60 was generated at Revalesio (Tacoma, WA) using a rotor/stator device, which incorporates controlled turbulence and Taylor-Couette-Poiseuille (TCP) flow. Briefly, sodium chloride (0.9%) for irrigation, USP pH 5.6 (4.5–7.0, Hospira), was processed at 4°C and a flow rate of 32 mL/s under 1 atm of oxygen back-pressure (7.8 mL/s gas flow rate) while maintaining a rotor speed of 3,450 rpm. The resulting fluid was immediately placed into glass bottles (KG- 33 borosilicate glass, Kimble-Chase) and sealed using gray chlorobutyl rubber stoppers (USP class 6, West Pharmaceuticals) to maintain pressure and minimize leachables. When tested after 24 h, the oxygen content was 55±5 ppm. Chemically, RNS60 contains water, sodium chloride, 50–60 parts/million oxygen, but no active pharmaceutical ingredients.

Following controls for RNS60 were also used in this study: a) NS, normal saline from the same manufacturing batch. This saline contacted the same device surfaces as RNS60 and was bottled in the same way, b) RNS10.3, saline that was processed through the same device without adding excess oxygen, and c) PNS60, saline with same oxygen content (55±5 ppm) that was prepared inside of the same device but was not processed with TCP flow. Careful analysis demonstrated that all three fluids were chemically identical.

### Analysis of RNS60, NS, RNS10.3, and PNS60 by liquid chromatography quadrupole time-of-flight mass spectrometry (LC-Q-TOF)

To test for compositional differences in RNS60, NS and PNS60, the LC-Q-TOF system was configured with an electrospray ionization interface (ESI) and the analysis performed in both positive and negative modes. The samples were introduced in triplicate via flow injection analysis (FIA) into the system and data acquisition performed in TOF mode (MS only, scan) in the 100 to 1000 *m/z* mass range. To facilitate visual comparison, the 100 to 1000 *m/z* scan range for each sample was separated into 9 segments of 100 *m/z* each and printed as part of the study data. The extracted segments from each of the two products were compared to the corresponding extracted segments for the source saline solution.

### RNS60 treatment

Mice were treated with RNS60 or NS (300 µL/mouse/d) from different phases of the disease via intraperitoneal (i.p.) injection. Statistical analysis was determined by the RS/1 multicomparison procedure using a one-way ANOVA and Dunnett's test for multiple comparisons with a common control group. Differences between means were considered significant when *p* values were less than 0.05.

### Histological microscopy

On 16 dpt (first chronic phase), five mice from each of the following groups (control, EAE, EAE+RNS60, and EAE+NS) were anesthetized. After perfusion with PBS (pH 7.4) and then with 4% (w/v) paraformaldehyde solution in PBS, cerebellum and whole spinal cord was dissected out from each mouse. The tissues were further fixed and then divided into halves: one-half was used for histological staining where as the other half was used for myelin staining as described earlier [13], [14], [15], [16]. For histological analysis, routine histology was performed to obtain perivascular cuffing and morphological details of CNS tissues of EAE mice. Paraformaldehyde-fixed tissues were embedded in paraffin, and serial sections (4 µm) were cut. Sections were stained with conventional H&E staining method. Digital images were collected under bright-field setting using an ×40 objective. Slides were assessed in a blinded fashion by three examiners for inflammation in different anatomical compartments (meninges and parenchyma). Inflammation was scored using the following scale as described: for meninges and parenchyma: 0, no infiltrating cells; 1, few infiltrating cells; 2, numerous infiltrating cells; and 3, widespread infiltration. For vessels: 0, no cuffed vessel; 1, one or two cuffed vessels per section; 2, three to five cuffed vessels per section and 3, more than five cuffed vessels per section. At least six serial sections of each spinal cord from each of five mice per group were scored and statistically analyzed by ANOVA.

### Staining for myelin

Sections were stained with Luxol fast blue for myelin as described earlier [15], [16]. Slides were assessed in a blinded fashion for demyelination by three examiners using the following scale: 0, normal white matter; 1, rare foci; 2, a few areas of demyelination; and 3, large areas of demyelination. At least six serial sections of each spinal cord from each of six mice per group were scored and statistically analyzed by ANOVA.

### Semi-quantitative RT-PCR analysis

Total RNA was isolated from splenic T cells and spinal cord by using the RNeasy mini kit (Qiagen) and from spleen and cerebellum by using the Ultraspec-II RNA reagent (Biotecx laboratories, Inc) following manufacturer's protocol. To remove any contaminating genomic DNA, total RNA was digested with DNase. Semi-quantitative RT-PCR was carried out as described earlier [15], [16] using a RT-PCR kit from Clonetech. Briefly, 1 µg of total RNA was reverse transcribed using oligo(dT)_12–18_ as primer and MMLV reverse transcriptase (Clontech) in a 20 µL reaction mixture. The resulting cDNA was appropriately-diluted, and diluted cDNA was amplified using Titanium Taq DNA polymerase and following primers. Amplified products were electrophoresed on a 1.8% agarose gels and visualized by ethidium bromide staining.

Foxp3: Sense, 5′-CAG CTG CCT ACA GTG CCC CTAG-3′

Antisense, 5′-CAT TTG CCA GCA GTG GGT AG-3′

CD25: Sense, 5′-AGC CAA GTA GGG TGT CTC TCA ACC-3′

Antisense, 5′-GCC CAG GAT ACA CAG TGA AGA ACG-3′

CD4: Sense, 5′- CCA ACA AGA GCT CAA GGA GAC CAC-3′

Antisense, 5′- CGT ACC CTC TTT CCT AGC AAA GGA-3′

CD62L: Sense, 5′- AGC CTC TTG CCA GCC AGG GT-3′

Antisense, 5′- CCA GCC CCG AGA ATG CGG TG-3′

CTLA4: Sense, 5′- GGT CCG GGT GAC TGT GCT GC-3′

Antisense, 5′- CCC GTT GCC CAT GCC CAC AA-3′

IFN-γ: Sense, 5′- GCTGTTACTGCCACGGCACA-3′

Antisense, 5′- GGACCACTCGGATGAGCTCA-3′

T-bet: Sense, 5′- GGAGCGGACCAACAGCATC-3′

Antisense, 5′- CCACGGTGAAGGACAGGAAT-3′

IL-10: Sense, 5′- GCACTGCTATGCTGCCTGCT-3′

Antisense, 5′- CCGATAAGGCTTGGCAACCC-3′

GATA3: Sense, 5′- TCTGGAGGAGGAACGCTAATGG-3′

Antisense, 5′- GAACTCTTCGCACACTTGGAGACTC-3′

IL-17: Sense, 5′- GCTGACCCCTAAGAAACCCC-3′

Antisense, 5′- GAAGCAGTTTGGGACCCCTT-3′

iNOS: Sense: 5′-CCCTTCCGAAGTTTCTGGCAGCAGC-3′

Antisense: 5′-GGCTGTCAGAGCCTCGTGGCTTTGG3′

IL-1β: Sense: 5′-CTCCATGAGCTTTGTACAAGG-3′

Antisense: 5′-TGCTGATGTACCAGTTGGGG-3′

MBP: Sense: 5′-TGGAGAGATTCACCGAGGAGA-3′

Antisense: 5′-TGAAGCTCGTCGGACTCTGAG-3′

CNPase: Sense: 5′-CTACCCTCCACGAGTGCAAGA-3′

Antisense: 5′-AGTCTAGTCGCCACGCTGTCT-3′

GAPDH: Sense: 5′-GGTGAAGGTCGGTGTGAACG3′

Antisense: 5′-TTGGCTCCACCCTTCAAGTG-3′

The relative expression of each gene with respect to GAPDH was measured after scanning the bands with a Fluor Chem 8800 Imaging System (Alpha Innotech, San Leandro, CA).

### Real-time PCR analysis

It was performed using the ABI-Prism7700 sequence detection system (Applied Biosystems) as described earlier [15], [16]. Briefly, reactions were performed in a 96-well optical reaction plates on cDNA equivalent to 50 ng DNase-digested RNA in a volume of 25 µL, containing 12.5 µL TaqMan Universal Master mix and optimized concentrations of FAM-labeled probe, forward and reverse primers following the manufacturer's protocol. All primers and FAM-labeled probes for mouse genes and GAPDH were obtained from Applied Biosystems. The mRNA expressions of respective genes were normalized to the level of GAPDH mRNA. Data were processed by the ABI Sequence Detection System 1.6 software and analyzed by ANOVA.

### Assay for NO synthesis

Synthesis of NO was determined by assay of culture supernatant for nitrite, a stable reaction product of NO with molecular oxygen, using ‘Griess’ reagent as described earlier [17], [18], [19].

### Assay of cytokines by ELISA

Supernatants were assayed for IFN-γ, IL-10 and IL-17 with high-sensitivity ELISA kits (BD Biosciences, Mountain View, CA) as described earlier [15], [20].

### Flow cytometry

Two-color flow cytometry was performed as described previously [17], [18]. Briefly, 1×10^6^ cells lymph node cells (LNC) or splenocytes suspended in flow staining buffer were incubated at 4°C with appropriately diluted FITC-labeled Ab to CD4 for 30 min, washed, and resuspended in fixation and permeabilization solution. Following incubation in dark for 30 min, cells were washed, blocked with test Fc block (anti-mouse CD16/32) in permeabilization buffer, and subsequently incubated with appropriately diluted PE-labeled Abs to T-bet, IFN-γ, GATA3, IL-4, IL-17, RORγT, or Foxp3 at 4°C in the dark. After incubation, the cell suspension was centrifuged, washed three times, and resuspended in an appropriate volume of flow staining buffer. The cells then were analyzed through FACS (BD Biosciences, San Jose, CA). Cells were gated based on morphological characteristics. Apoptotic and necrotic cells were not accepted for FACS analysis.

### Assay of suppressive activity of Tregs

Because cells from tomato red transgenic (B6.129(Cg)-Gt(ROSA)26Sor^tm4(ACTB-tdTomato,-EGFP)Luo^/J) mice exhibit red color, we used these mice (Jackson Laboratories, Bar Harbor, ME) for clear visualization of the suppressive activity of Tregs. These mice were immunized with MOG (100 µg/mouse) suspended in IFA containing 60 µg *M. tuberculosis* and 12 d after immunization, splenocytes were isolated and re-primed with MOG (10 µg/ml) for 2 d. These MOG-primed tomato red T cells expressed Th1 and Th17 cytokines (data not shown). In a parallel experiment, B6.129 mice were also immunized with MOG and splenocytes were re-primed with MOG in the presence of RNS60 (10% v/v) for 2 d followed by purification of CD4+CD25+ Tregs. Then these RNS60-induced Tregs were added to MOG-primed splenocytes isolated from tomato red transgenic mice at a ratio of 2∶1 of tomato red T cell:RNS60-induced Tregs and the suppressive activity of Tregs was monitored by the inhibitory effect on the IFN-γ expression by MOG-primed tomato red T cells. Therefore, after 24 h of incubation, CD3+ T cells were purified and immunostained for IFN-γ (green). IFN-γ-expressing red cells were counted and expressed as percent of total red cells.

## Results

### Compositions of different saline solutions

In order to detect any potential chemical contaminants, RNS60 was carefully compared with two control solutions: a) NS, unprocessed normal saline from the same manufacturing batch that contacted the same device surfaces as RNS60 and was bottled in the same way; and b) PNS60, normal saline from the same manufacturing batch that was prepared inside of the same device and bottled in the same way, but was not processed with the TCP flow. PNS60 contained a level of oxygen comparable to RNS60 (50–60 parts/million).

Careful ICP-MS testing for 26 metals and TOC analysis revealed no differences between RNS60 and the control solutions within detection limits (data not shown). Similarly, triplicate MS-TOF analysis with and without chromatographic pre-treatment, using either negative or positive ion modes, did not reveal chemical composition differences in the 100–1000 m/e range (only a part of it has been presented in Figure S1). In all cases, samples for the fluid product and saline source solution were found to be identical within approximately 1 ppb.

### Enrichment of the Treg population by RNS60

**Figure 1:**
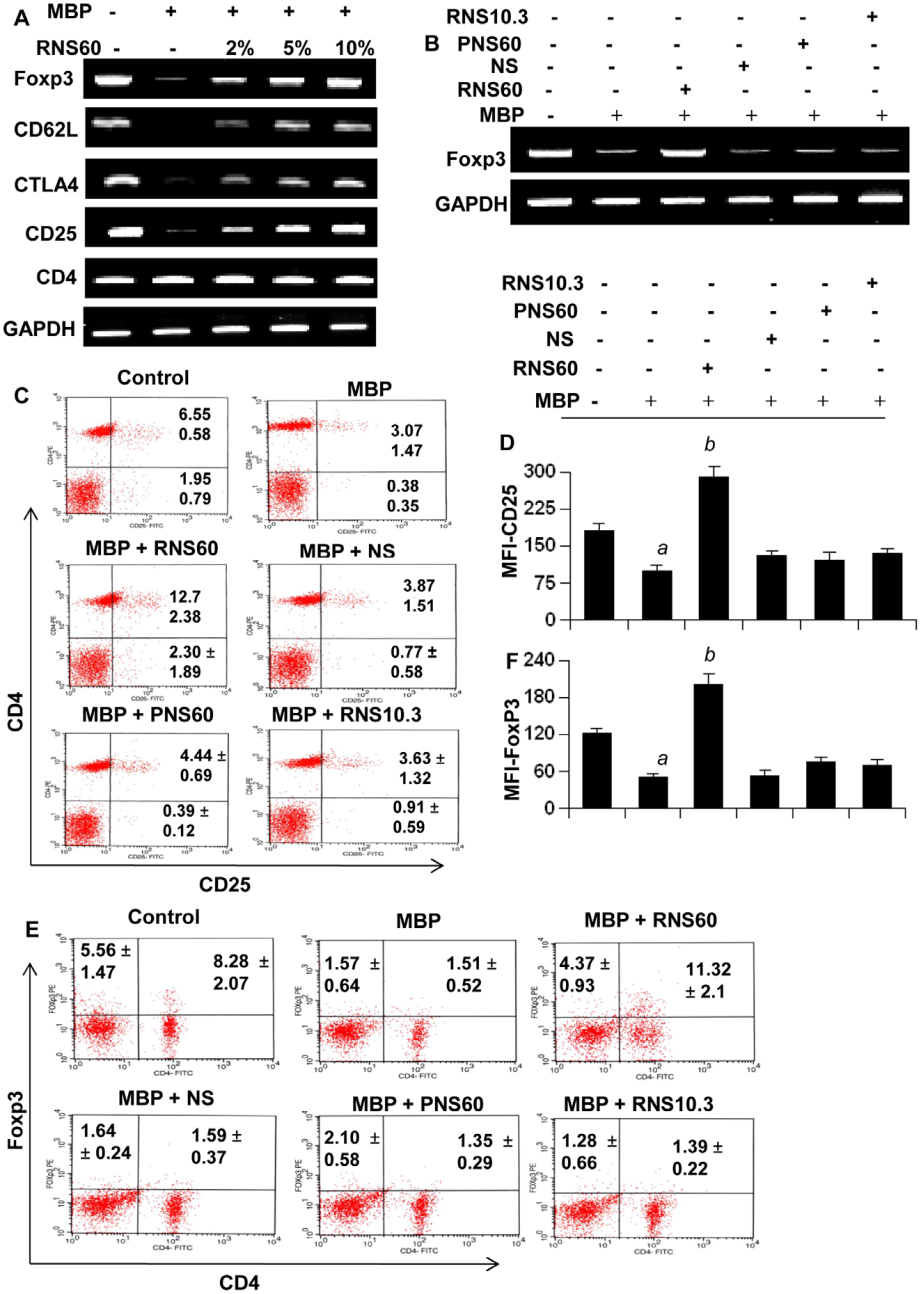
Enrichment of Tregs by RNS60, but not NS, PNS60 and RNS10.3. A) Lymph node cells (LNC) isolated from MBP-immunized donor mice were stimulated with MBP in the presence of different concentrations of RNS60 for 24 h followed by monitoring the mRNA expression of Foxp3, CD62L, CTLA4, CD25, and CD4 by semi-quantitative RT-PCR. B) LNC from MBP-immunized mice were stimulated with MBP in the presence of 10% (v/v) of RNS60, NS, RNS10.3, or PNS60 for 24 h followed by monitoring the mRNA expression of Foxp3. C) LNC isolated from MBP-immunized mice were stimulated with MBP in the presence of 10% (v/v) of RNS60, NS, RNS10.3, and PNS60 for 24 h followed by FACS analysis using appropriately diluted PE-conjugated anti-CD25 and FITC-conjugated anti-CD4 Abs. D) The MFI of CD25 in CD4+ population was calculated by using CellQuest software. Data are mean ± SD of three different experiments. *^a^p<0.001* vs control; *^b^p<0.001* vs MBP. E) LNC from MBP-immunized mice were stimulated with MBP in the presence of 10% (v/v) of RNS60, NS, RNS10.3, and PNS60 for 24 h followed by FACS analysis using appropriately diluted PE-conjugated anti-Foxp3 and FITC-conjugated anti-CD4 Abs. F) The MFI of Foxp3 in CD4+ population was calculated by using CellQuest software. Data are mean ± SD of three different experiments. *^a^p<0.001* vs control; *^b^p<0.001* vs MBP.

Because Tregs are most important immunomodulatory subtype of T lymphocytes, in order to understand immunomodulatory effect of RNS60, at first, we examined the effect of RNS60 on Tregs. Earlier we have demonstrated that antigen priming is capable of suppressing Tregs [17]. Therefore, MBP-primed lymph node cells (LNC) were re-primed with MBP in the presence or absence of different doses of RNS60, NS, RNS10.3, and PNS60 followed by monitoring the expression of the regulatory T cell marker Foxp3. As expected, MBP-priming led to marked loss of Foxp3 (Fig. 1A). However, RNS60 dose-dependently increased the expression of Foxp3 in MBP-primed LNC (Fig. 1A). On the other hand, normal saline (NS), TCP-modified saline without excess oxygen (RNS10.3) and saline containing excess oxygen without the TCP modification (PNS60) had no such on the expression of Foxp3 (Fig. 1B), suggesting the specificity of the effect. Because Foxp3+ T cells usually express CD25, CTLA4 and CD62L, we also analyzed the mRNA expression of these molecules. Similar to Foxp3, the mRNA expression of CD25, CD62L and CTLA4 also decreased upon MBP-priming and RNS60 rescued the expression of these molecules as evident from our semi-quantitative RT-PCR (Fig. 1A). On the other hand, either MBP-priming or RNS60 treatment had no effect on the mRNA expression of CD4 (Fig. 1A), suggesting that these results are not due to any alteration in CD4+ T cells.

As most of the Foxp3+ T cells are phenotypically characterized by surface co-expression of CD4 and CD25 [2], [21], [22], we analyzed the effect of RNS60 on the proportion of CD4+CD25+ T cell population. As expected, there was a significant reduction in CD4+CD25+ population of T cells by MBP-priming as evident from FACS dot plot (Fig. 1C) and mean fluorescence intensity (MFI) (Fig. 1D). However, RNS60, but not NS, PNS60 or RNS10.3, strongly increased the CD4+CD25+ population in MBP-primed LNC (Fig. 1C–D). We believe that the increase in CD25 is because of upregulation of Foxp3 by RNS60. However, it is possible that other T cell populations are altered by RNS60 treatment giving the impression that the Foxp3+ T cells are increasing. To rule out this possibility, we performed intracellular staining of Foxp3 along with surface staining for CD4. As evident from figure 1E, treatment of MBP-primed LNC with RNS60, but not NS, PNS60 or RNS10.3, led to increase Foxp3+ T cells. MFI calculation in figure 1F also shows that RNS60 treatment resulted in significant increase in Foxp3.

**Figure 2:**
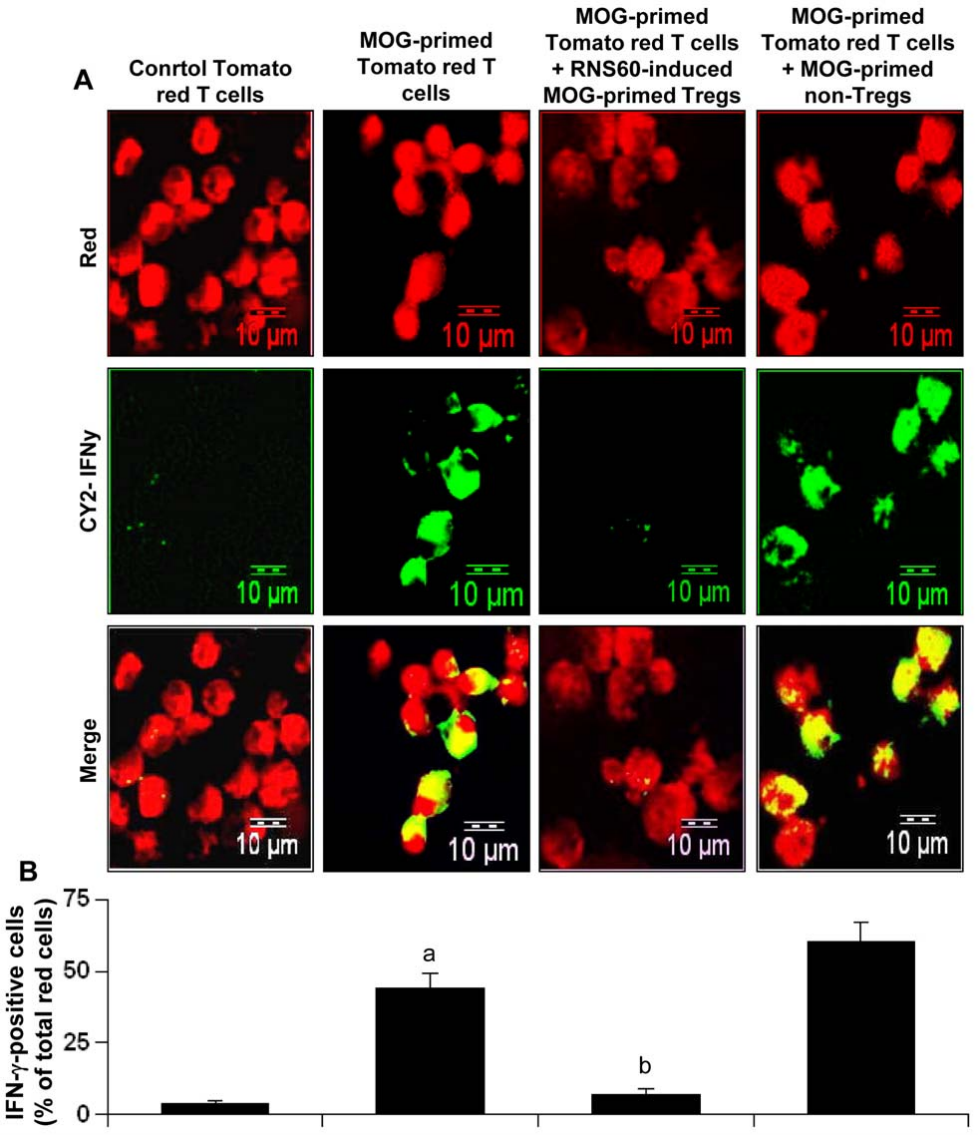
Suppressive activity of RNS60-induced Tregs. A) Tomato red transgenic mice were immunized with MOG (100 µg/mouse) and 12 d after immunization, splenocytes were isolated and re-primed with MOG (10 µg/ml) for 2 d. Similarly, B6.129 mice were also immunized with MOG and splenocytes were re-primed with MOG in the presence of RNS60 (10% v/v) for 2 d followed by purification of CD4+CD25+ Tregs. Then these Tregs were added to MOG-primed splenocytes isolated from tomato red transgenic mice at a ratio of 2∶1 of tomato red T cell:RNS60-induced Tregs. After 24 h, CD3+ T cells were purified and immunostained for IFN-γ (green). Cells from tomato red transgenic mice exhibited red color. B) IFN-γ-expressing red cells were counted and expressed as percent of total red cells. Data are mean ± SEM of 20 different images. *^a^p<0.001* vs control tomato red T cells; *^b^p<0.001* vs MOG-primed tomato red T cells.

To further confirm that RNS60 does in fact augment Tregs, we examined the suppressive activity of RNS60-induced Tregs using a new assay technique that involves tomato red transgenic mice. As evident from Figure 2A and 2B, IFN-γ expression was very low in normal tomato red T cells and MOG priming significantly increased the expression of IFN-γ in these T cells. However, RNS60-induced MOG-primed Tregs of B6.129 mice markedly suppressed the expression of IFN-γ in MOG-primed tomato red T cells (Fig. 2A & 2B). This result was specific as MOG-primed non-Tregs increased the expression of IFN-γ in tomato red T cells. These results demonstrate that RNS60-induced Tregs are functionally active.

### How does RNS60 enrich Tregs?

**Figure 3:**
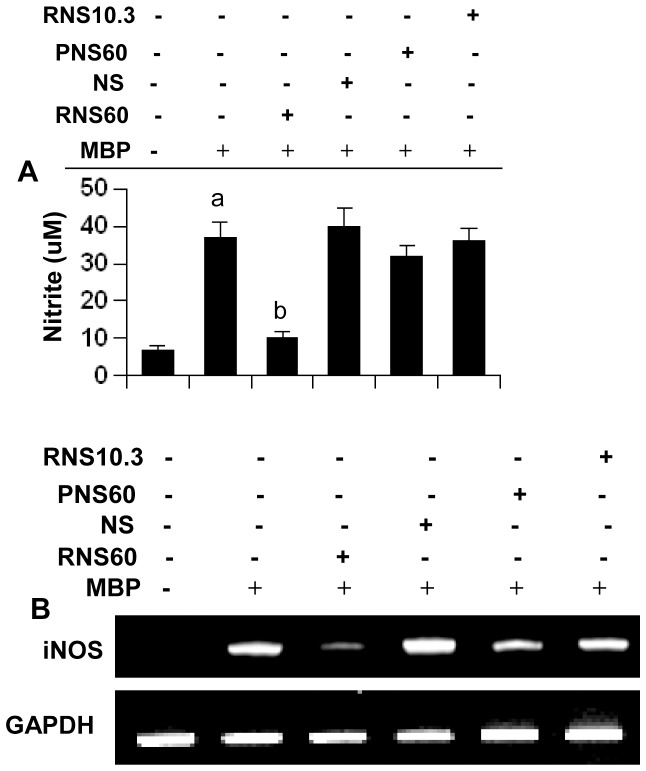
Effect of RNS60, NS, PNS60, and RNS10.3 on the expression of iNOS in MBP-primed LNC. LNC isolated from MBP-immunized mice were stimulated with MBP for 24 h in the presence of 10% (v/v) of RNS60, NS, RNS10.3, or PNS60 followed by monitoring the level of nitrite (A) and the expression of iNOS mRNAs by semi-quantitative RT-PCR (B). Results are mean ± SD of three different experiments. *^a^p*<0.0001 versus control; *^b^p*<0.0001 versus MBP.

Recently, we have reported that NO is a critical regulator of Tregs [17]. While increasing the level of NO decreases Tregs, reducing the level of NO enriches Tregs [17]. Therefore, we were prompted to investigate whether RNS60 increased the number of Tregs via decreasing NO. At first, we examined whether RNS60 would inhibit NO production in MBP-primed LNC and splenocytes. As expected, MBP priming alone induced the production of NO in LNC (Fig. 3A). However, RNS60 strongly suppressed the induction of NO production (Fig. 3A). Although at lower concentrations (1 and 2% v/v), RNS60 was not effective in inhibiting the production of NO (data not shown), at higher concentrations, RNS60 markedly suppressed the induction of NO production as evident from the estimation of nitrite (Fig. 3A). On the other hand, NS, RNS10.3 or PNS60 had no such inhibitory effect on the production of NO (Fig. 3A). To understand the mechanism further, we investigated the effect of RNS60 on mRNA level of iNOS in MBP-primed LNC. It is evident from semi-quantitative RT-PCR analysis that RNS60, but not NS, RNS10.3 or PNS60, inhibited the mRNA expression of iNOS in MBP-primed LNC (Fig. 3B). Similarly, RNS60, but not NS, also suppressed the induction of NO production (Figure S2A) and the mRNA expression of iNOS (Figure S2B for RT-PCR and Figure S2C for real-time PCR) in MBP-primed splenocytes. Together, these results suggest that RNS60 is capable of suppressing the expression of iNOS in MBP-primed LNC and splenocytes. Next we monitored the level of Foxp3 mRNA. In contrast to iNOS, MBP-priming decreased the mRNA expression of Foxp3, whereas RNS60 blocked the loss of Foxp3 in MBP-primed splenocytes (Figure S2B for RT-PCR and Figure S2C for real-time PCR).

**Figure 4:**
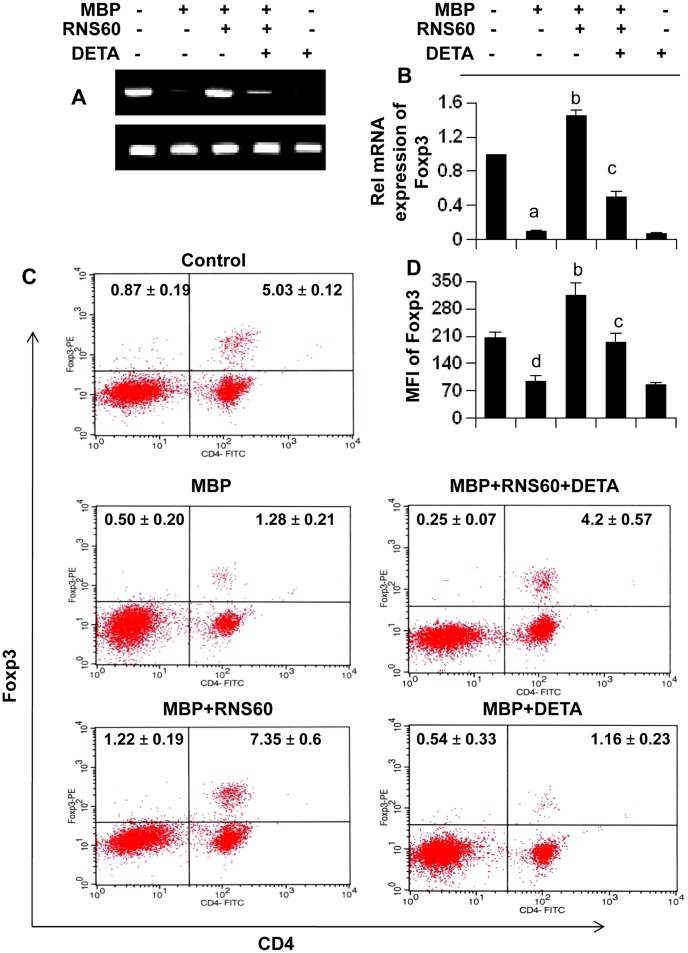
NO abrogates RNS60-mediated upregulation of Foxp3 in MBP-primed splenocytes. Splenocytes isolated from MBP-immunized mice were stimulated with MBP for 24 h in the presence or absence of 10% (v/v) RNS60 and different concentrations of DETA-NONOate followed by monitoring the expression of Foxp3 by semi-quantitative RT-PCR (A) and real-time PCR (B) and FACS analysis (C). The MFI of Foxp3 (D) in CD4+ population was calculated by using CellQuest software. Data are mean ± S.D. of three different experiments. *^a^p<0.001* vs control; *^b^p<0.001* vs MBP; *^c^p<0.001* vs (MBP+RNS60); *^d^p<0.05* vs control.

Next, to directly test a role of NO in RNS60-mediated modulation of Foxp3, we added DETA-NONOate (an NO donor) to RNS60-treated splenocytes. It is evident from RT-PCR (Fig. 4A), real-time PCR (Fig. 4B), FACS dot plot (Fig. 4C), and MFI analysis (Fig. 4D) that RNS60 increases the level of Foxp3 in MBP-primed splenocytes. However, this increase and/or protection of Foxp3 mRNA and protein was completely abrogated by DETA-NONOate treatment (Fig. 4A–D), indicating an important role of NO in RNS60-mediated upregulation of Foxp3 and enrichment of Tregs.

### Suppression of the Th17 response by RNS60

**Figure 5:**
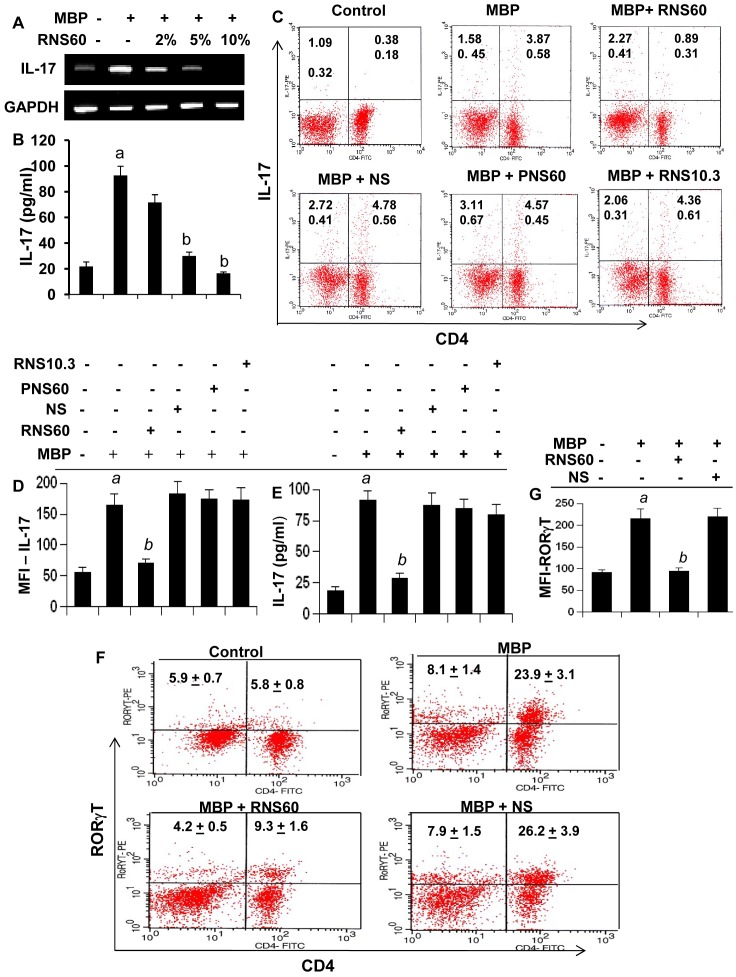
Suppression of Th17 cells by RNS60, but not NS, PNS60 and RNS10.3. LNC isolated from MBP-immunized donor mice were stimulated with MBP in the presence of different concentrations of RNS60 for 48 h followed by monitoring the mRNA expression of IL-17 by semi-quantitative RT-PCR (A) and the protein level of IL-17 in supernatants by ELISA (B). Data are mean ± S.D. of three different experiments. *^a^p<0.001* vs control; *^b^p<0.001* vs MBP. LNC were stimulated with MBP in the presence of 10% v/v of RNS60, NS, PNS60, or RNS10.3. C) After 72 h of stimulation, T cells were incubated with appropriately diluted PE-conjugated anti-IL-17 and FITC-conjugated anti-CD4 Abs followed by FACS analysis. The percentage of relevant cells is indicated in their respective quadrants. D) The MFI of IL-17 in CD4+ population was calculated by using CellQuest software. E) Supernatants were assayed for IL-17 by ELISA. Data are mean ± SD of three different experiments. *^a^p<0.001* vs control; *^b^p<0.001* vs MBP. F) LNC isolated from MBP-immunized donor mice were stimulated with MBP in the presence of 10% v/v of RNS60 or NS followed by FACS analysis using appropriately-diluted PE-conjugated anti-RORγT and FITC-conjugated anti-CD4 Abs. G) The MFI of RORγT in CD4+ population was calculated by using CellQuest software. Data are mean ± SD of three different experiments. *^a^p<0.001* vs control; *^b^p<0.001* vs MBP.

After the discovery of IL-23, Th17 cells are considered to play a more active role than Th1 cells in the disease process of EAE and MS [23]. It has been found that there is an inverse relationship between Th17 cells and Tregs [24]. Because RNS60 enriched Tregs, we examined whether RNS60 was capable of regulating Th17 cells. While MBP-priming increased the expression of IL-17 mRNA (Fig. 5A), protein (Fig. 5B) and the level of CD4+IL-17+ T cells in LNC (Fig. 5C), RNS60 markedly suppressed the MBP-induced upregulation of IL-17 mRNA and protein as well as the CD4+IL-17+ T cell population (Fig. 5A–C). On the other hand, saline controls (NS, PNS60 and RNS10.3) had no such suppressive effects on CD4+IL-17+ T cell (Fig. 5C). MFI analysis of IL-17 (Fig. 5D) within the CD4+ population also supported this finding. We also monitored the production of IL-17 from LNC. As evident from Figure 5E, MBP-priming increased the production of IL-17 in LNC and RNS60, but not NS, PNS60 or RNS10.3, suppressed MBP-induced IL-17 production. Th17 cells are also characterized by a transcription factor called RORγT [25]. To confirm the regulation of Th17 cells, we also monitored RORγT by intracellular FACS staining. Consistent with the regulation of CD4+IL-17+ T cells, MBP-priming increased the level of CD4+RORγT+ T cells in LNC (Fig. 5F) and RNS60, but not NS, markedly suppressed MBP-induced upregulation of CD4+RORγT+ T cells (Fig. 5F). This is also corroborated by MFI analysis of RORγT within the CD4+ population (Fig. 5G). These results clearly show that RNS60 is capable of suppressing Th17 cells.

### Switching of Th1 to Th2 in response to RNS60

Similar to Th17 cells, Th1 cells are also autoimmune inflammatory and switching of the Th1 to a Th2 phenotype is one of the ways to ameliorate the disease [11], [23], [26]. Because RNS60 increased Tregs, which are known to suppress Th1 cells via releasing TGF-β and IL-10, we examined whether RNS60 was capable of suppressing the autoimmune Th1 response. While T-bet-dependent IFN-γ production is a characteristic of Th1 cells, Th2 cells display GATA3-dependent IL-10 and IL-4 release [10], [27], [28]. As expected, MBP-priming increased the level of CD4+IFN-γ+ T cells (Figure S3A&B). However, RNS60, but not NS, PNS60 or RNS10.3, suppressed CD4+IFN-γ+ T cells in MBP-primed LNC (Figure S3A&B). Similarly, MBP-priming induced the production of IFN-γ protein in LNC and RNS60, but not NS, inhibited MBP-induced IFN-γ production (Figure S3C). To confirm this finding further, we performed intracellular FACS analysis for T-bet. While MBP-priming increased the level of CD4+T-bet+ T cells, RNS60, but not NS, markedly suppressed MBP-induced upregulation of T-bet in CD4+ T cells (Figure S3D&E). RT-PCR analysis of LNC for IFN-γ and T-bet (Figure S3F) also supports this finding.

**Figure 6:**
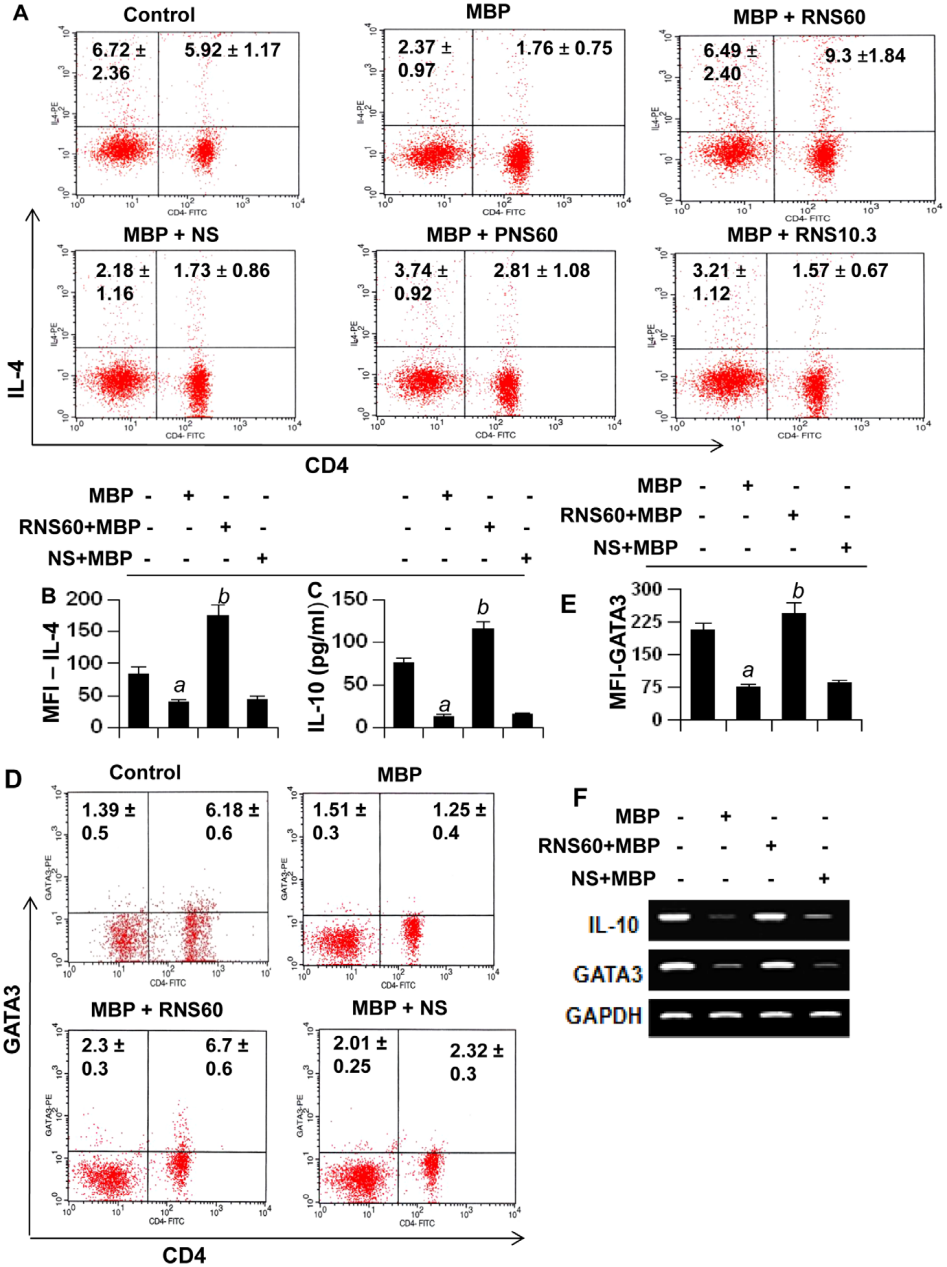
Regulation of Th2 cells by RNS60, NS, PNS60, and RNS10.3. LNC isolated from MBP-immunized donor mice were stimulated with MBP in the presence of 10% v/v of RNS60, NS, PNS60, or RNS10.3. A) After 72 h of stimulation, T cells were incubated with appropriately diluted PE-conjugated anti-CD4 and FITC-conjugated anti-IL-4 Abs followed by FACS analysis. The percentage of relevant cells is indicated in their respective their respective quadrants. B) The mean fluorescence intensity (MFI) of IL-4 in CD4+ population was calculated by using CellQuest software. C) Supernatants were assayed for IL-10 by ELISA. Data are mean ± SD of three different experiments. *^a^p<0.001* vs control; *^b^p<0.001* vs MBP. D) LNC isolated from MBP-immunized donor mice were stimulated with MBP in the presence or absence of RNS60 (10% v/v) and NS (10% v/v), respectively followed by FACS analysis with appropriately diluted PE-conjugated anti-GATA3 and FITC-conjugated anti-CD4 Abs. The percentage of relevant cells is indicated in their respective quadrants. E) The MFI of GATA3 in CD4+ population was calculated by using CellQuest software. Data are mean ± SD of three different experiments. *^a^p<0.001* vs control; *^b^p<0.001* vs MBP. F) LNC isolated from MBP-immunized donor mice were stimulated with MBP in the presence or absence of RNS60 or NS, for 48 h followed by monitoring the mRNA expression of IL-10 and GATA3 by semi-quantitative RT-PCR. Results represent three independent experiments.

Next, we analyzed the Th2 responses by monitoring Th2 cytokines (IL-4 and IL-10). As evident from Figure 6A&B, MBP-priming suppressed CD4+IL-4+ T cells in MBP-primed LNC. However, RNS60 markedly increased intracellular the level of CD4+IL-4+ T cells in MBP-primed LNC (Fig. 6A&B). This effect was specific as NS, PNS60 or RNS10.3 had no effect. Similarly, MBP-priming strongly inhibited the production of IL-10 from LNC, and RNS60, but not NS, abrogated MBP-induced loss and increased the level of IL-10 (Fig. 6C). To confirm this finding further, we monitored the level of the Th2 signature transcription factor GATA3. As apparent from Figure 6D&E, MBP-priming decreased CD4+GATA3+ T cell population in LNC and RNS60, but not NS, markedly increased the level of CD4+GATA3+ T cells in MBP-primed LNC. RT-PCR analysis of LNC for IL-10 and GATA3 (Fig. 6F) also supports this finding. Together, these results suggest that RNS60 is capable of suppressing the Th1 response, while augmenting the Th2 response.

### RNS60 inhibits clinical symptoms and disease severity of EAE in female SJL/J mice

**Figure 7:**
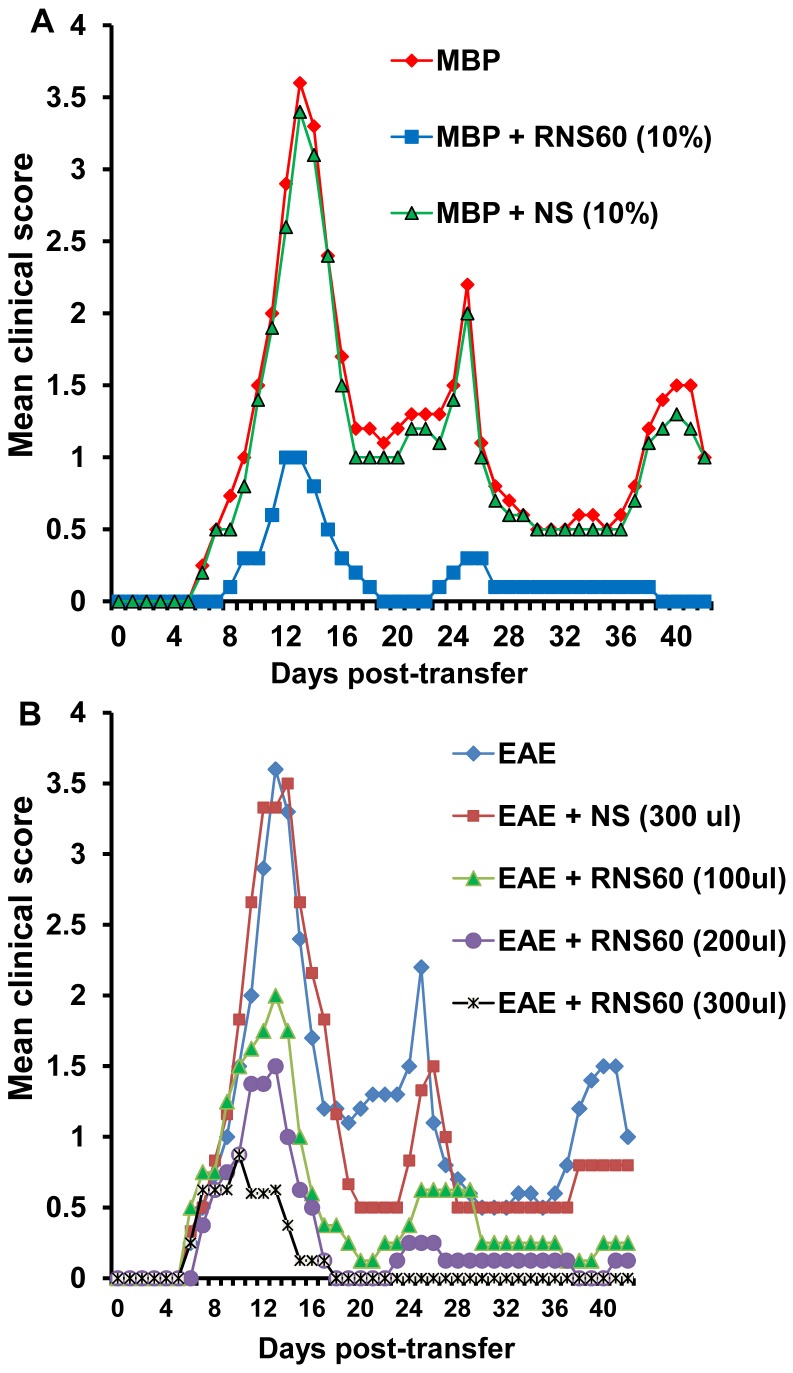
Effect of RNS60 on clinical symptoms of adoptively-transferred relapsing-remitting EAE in mice and encephalitogenicity of MBP-primed T cells. A) EAE was induced in female SJL/J mice by adoptive transfer of MBP-primed T cells. From 0 dpt, mice were treated with different doses of RNS60 or NS via i.p. injection (dpt 1–8, alternate day; dpt 9–16, daily; dpt 17 onwards, alternate day). Five mice were included in each group. Mice (n = 5) were examined for clinical symptoms daily until 42 dpt. B) MBP-primed T cells isolated from female SJL/J donor mice were treated with either RNS60 or NS during MBP re-priming for 4 d followed by tail vein injection of MBP-primed T cells into naïve female SJL/J mice. Five mice were included in each group.

We examined whether i.p. administration of RNS60 can inhibit the clinical symptoms and disease severity in adoptively-transferred EAE mice. Multiple groups of mice were treated with different doses of RNS60 from 0 days post transfer (dpt) of activated cells. An additional group of mice was treated with unprocessed normal saline (NS) from the same manufacturing batch used to generate RNS60 as a negative control. Clinical scores were observed on each day after transfer. Since the relapsing-remitting type of EAE is associated with multiple chronic phase peaks following the acute phase peak, we continued our observations until 42 dpt. At a dose of 100 µL/mouse, RNS60 significantly inhibited clinical symptoms (Fig. 7A & Table 1) without reducing disease incidence. On the other hand, at a dose of 300 µL/mouse, a dramatic inhibition of clinical symptoms and a significant reduction in disease incidence were observed in acute as well as chronic phases of EAE (Fig. 7A). Only piloerection was observed as the highest clinical symptom in most of the mice that received RNS60 at a dose of 300 µL/mouse (Fig. 7A & Table 1). On the other hand, NS remained unable to inhibit the clinical symptoms of EAE (Fig. 7A & Table 1), suggesting the specificity of the effect. These findings demonstrate that RNS60 is capable of inhibiting clinical symptoms and disease severity in acute as well as chronic phases of EAE in a dose-dependent manner.

**Table 1:** Effect of RNS60 and NS on clinical symptoms of EAE.

Treatment	Incidence	Mean peak clinical score	Suppression of EAE	
			Incidence	Score
EAE	6/6	3.6		
EAE+RNS60 (100 µl/mouse/d)	5/5	2.0	0	44^b^
EAE+RNS60 (200 µl/mouse/d)	4/5	1.5	20	58^a^
EAE+RNS60 (300 µl/mouse/d)	2/5	0.8	60^a^	78^a^
EAE+NS (300 µl/mouse/d)	5/5	3.5	0	3

EAE was induced in female SJL/J mice through adoptive transfer of MBP-primed T cells. From 0 dpt, mice were treated with different doses of RNS60 or NS via i.p. injection (dpt 1–8, alternate day; dpt 9–16, daily; dpt 17 onwards, alternate day). While a clinical score of 1 was considered as the incidence of EAE in mice, a clinical score of 0 was considered to be normal.

a *p*<0.001,

b *p*<0.01 vs EAE (control).

### RNS60 inhibits encephalitogenicity of MBP-primed T cells

As MBP-primed T cells are encephalitogenic, and adoptive transfer of these T cells induces EAE in recipient mice, we investigated whether RNS60 was capable of inhibiting encephalitogenicity of MBP-primed T cells. In order to test this, T cells isolated from MBP-immunized donor mice were cultured with MBP in the presence or absence of 10% RNS60 or NS for 4 days. Untreated, RNS60-treated, and NS treated MBP-primed T cells were then adoptively transferred to recipient mice. Our results showed that mice receiving RNS60-treated MBP-primed T cells exhibited significantly reduced clinical symptoms and disease severity compared to mice receiving either untreated or NS-treated cells (Fig. 7B). These results suggest that RNS60 inhibits the encephalitogenicity of MBP-primed T cells.

### Therapeutic administration of RNS60 inhibits progression of adoptively-transferred EAE

**Figure 8:**
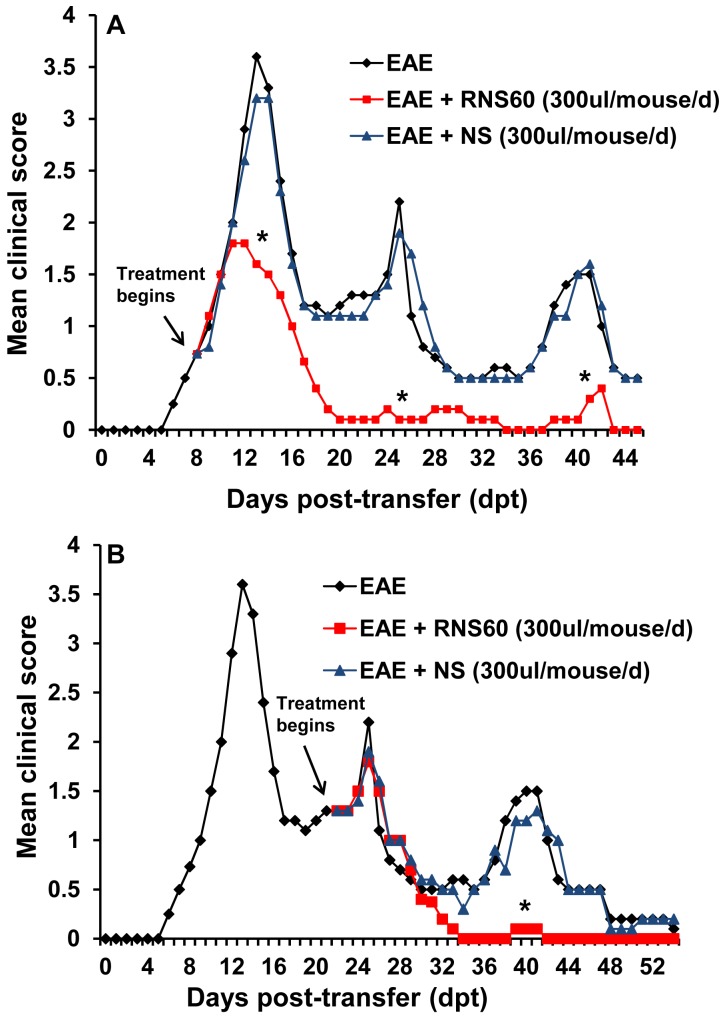
RNS60 inhibits the progression of adoptively-transferred relapsing-remitting EAE in mice. EAE was induced in female SJL/J mice by adoptive transfer of MBP-primed T cells. A) From the onset of acute phase (8 dpt), mice were treated with either RNS60 or NS via i.p. injection (dpt 8–16, daily; dpt 17 onwards, alternate day). Mice (n = 5) were examined for clinical symptoms everyday until 45 dpt. B) From the onset of relapsing-remitting phase (22 dpt), mice were treated with either RNS60 or NS via i.p. injection (alternate day). Mice (n = 5) were examined for clinical symptoms daily until 54 dpt.

To test whether RNS60 would also inhibit the disease progression in adoptively-transferred EAE, mice were treated with RNS60 in two different groups. In the first group, mice were treated with RNS60 from the onset of acute phase (8 dpt). An inhibitory effect of RNS60 on the clinical symptoms was clearly observed within 4 days of treatment (from 12 dpt, Fig. 8A). Greater inhibition was observed on subsequent days of treatment, which was maintained throughout the duration (45 dpt) of the experiment (Fig. 8A). On the other hand, NS had no such inhibitory effect (Fig. 8A). In the second group, RNS60 treatment began from the onset of relapsing phase (22 dpt) and was continued until 54 dpt. Here, too, RNS60, but not NS, halted disease progression (Fig. 8B). However, in contrast to the first instance, the inhibitory effect of RNS60 was manifested after 10 days of treatment (32 dpt). The EAE disease severity in the RNS60-treated group was close to 0 (normal) from 32 dpt until the end of the study (54 dpt) (Fig. 8B). These results clearly demonstrate that RNS60 can ameliorate the ongoing relapsing-remitting EAE when administered either early (at the onset of acute disease) or late (at the onset of relapsing disease).

### RNS60 inhibits infiltration of mononuclear cells, inflammation and demyelination in the CNS of EAE

**Figure 9:**
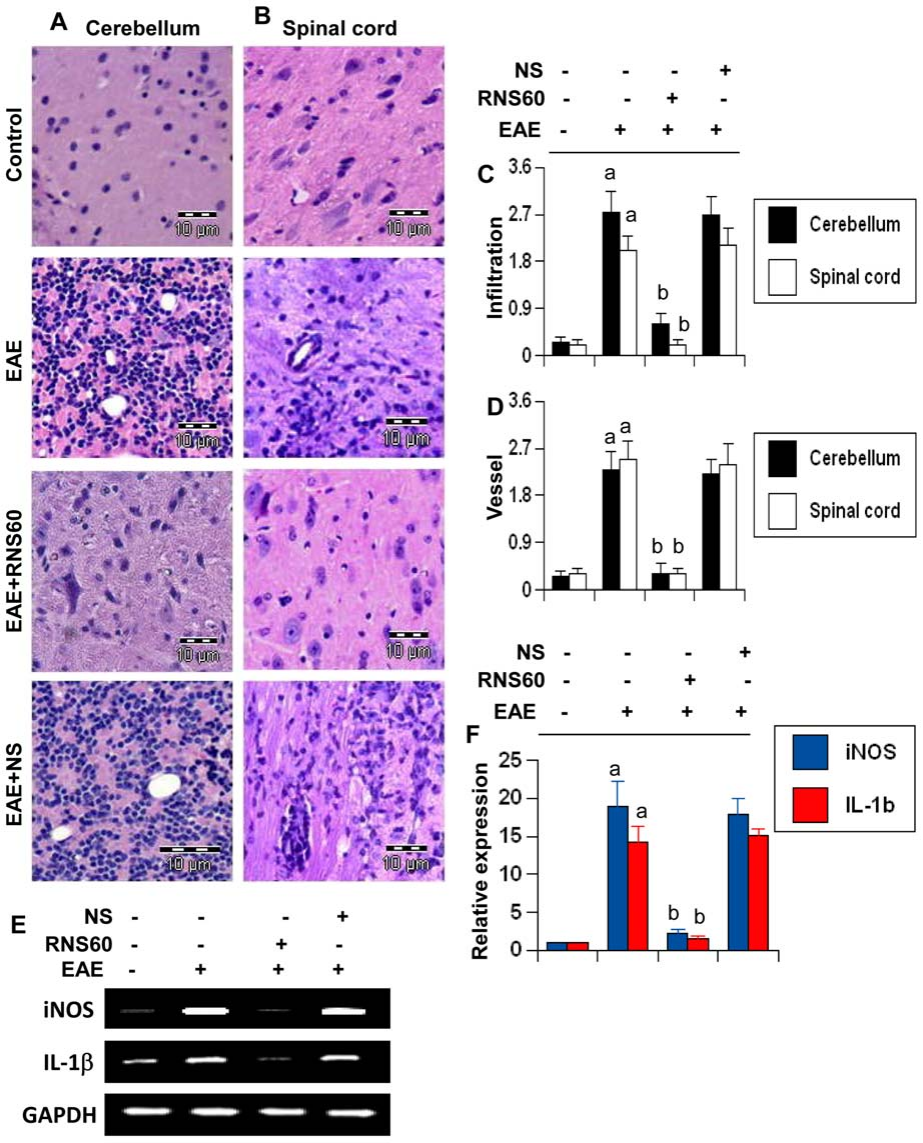
RNS60 inhibits infiltration of mononuclear cells and inflammation in the CNS of EAE mice. Cerebellar (A) and spinal cord (B) sections of control EAE (16 dpt), and either RNS60− or NS-treated EAE mice (16 dpt receiving RNS60/NS from 8 dpt) were stained with H & E. Digital images were collected under bright field setting using a 40× objective. Infiltration (C) and cuffed vessel (D) were represented quantitatively by using a scale as described in materials and methods. Data are expressed as the mean ± SEM of five different mice. *^a^p<0.001* vs control; *^b^p<0.001* vs EAE. Cerebellum of control, EAE and either RNS60− or NS-treated EAE mice was analyzed for iNOS and IL-1β by semi-quantitative RT-PCR (E) and quantitative real-time PCR (F). Data are expressed as the mean ± SEM of five different mice. *^a^p<0.001* vs control; *^b^p<0.001* vs EAE.

EAE as well as MS is caused by infiltration of autoreactive T cells and associated mononuclear cells, like macrophages, into the CNS, followed by broad-spectrum inflammatory events [10], [29]. We examined whether RNS60 attenuated infiltration and inflammation in adoptively-transferred EAE mice. Mice receiving RNS60 from 8 dpt (onset of the acute phase) were sacrificed on 16 dpt. H & E staining showed widespread infiltration of inflammatory cells into cerebellum (Fig. 9A) and spinal cord (Fig. 9B) of EAE mice. On the other hand, RNS60 treatment markedly inhibited the infiltration of inflammatory cells into both cerebellum and spinal cord of EAE mice (Fig. 9A&B). In contrast, NS was unable to inhibit the infiltration of inflammatory cells (Fig. 9A&B). Quantitation of the relative level of inflammatory cells showed that RNS60, but not NS, dramatically reduced infiltration (Fig. 9C) and the appearance of cuffed vessels (Fig. 9D) in cerebellum and spinal cord of RR-EAE mice.

Since infiltration was inhibited, we next examined whether RNS60 was capable of inhibiting the expression of proinflammatory molecules in the CNS of EAE mice. Marked expression of pro-inflammatory molecules like iNOS and IL-1β was observed in the cerebellum of untreated EAE mice compared to control mice (Fig. 9E & F). However, RNS60 treatment dramatically reduced the expression of these pro-inflammatory molecules in the cerebellum of EAE mice (Fig. 9E & F).

**Figure 10:**
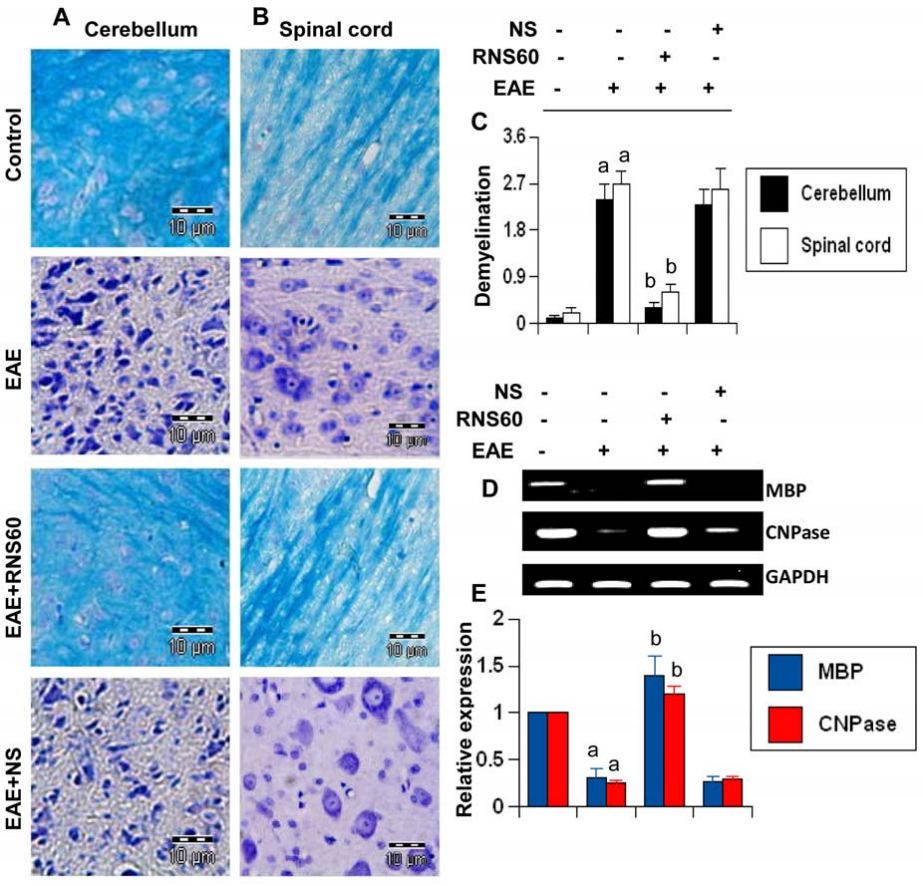
RNS60 inhibits demyelination in the CNS of EAE mice. Cerebellar (A) and spinal cord (B) sections of control EAE (16 dpt), and either RNS60− or NS-treated EAE mice (16 dpt receiving RNS60/NS from 8 dpt) were stained with Luxol fast blue. Digital images were collected under bright field setting using a 40× objective. (C) Demyelination was represented quantitatively by using a scale as described in materials and methods. Data are expressed as the mean ± SEM of five different mice. *^a^p<0.001* vs control; *^b^p<0.001* vs EAE. Cerebellum of control, EAE and either RNS60− or NS-treated EAE mice was analyzed for MBP and CNPase by semi-quantitative RT-PCR (D) and quantitative real-time PCR (E). Data are expressed as the mean ± SEM of five different mice. *^a^p<0.001* vs control; *^b^p<0.001* vs EAE.

It is believed that infiltration of blood mononuclear cells and associated neuroinflammation plays an important role in CNS demyelination observed in MS patients and EAE animals [10], [30], [31]. Therefore, we examined whether RNS60 protected EAE mice from demyelination. We stained cerebellar and spinal cord sections by luxol fast blue (LFB) for myelin and observed widespread demyelination zones in the white matter (Fig. 10A–C). However, RNS60 treatment remarkably restored myelin level in cerebellum and spinal cord of RR-EAE mice (Fig. 10A–C). In contrast, NS was unable to restore myelin level in CNS tissues of EAE mice (Fig. 10A–C). To confirm this finding, we monitored the expression of two myelin genes, MBP and CNPase, and observed a marked loss of mRNA expression of these genes in the cerebellum of untreated EAE mice compared to control mice (Fig. 10D & E). A significant restoration of myelin gene mRNA expression was observed in EAE mice that were treated with RNS60, but not in mice treated with NS (Fig. 10D & E). Taken together, these results demonstrate that RNS60 inhibits infiltration of mononuclear cells, inflammation, and demyelination in the CNS of EAE mice.

### Immunomodulation in EAE mice by RNS60 treatment

**Figure 11:**
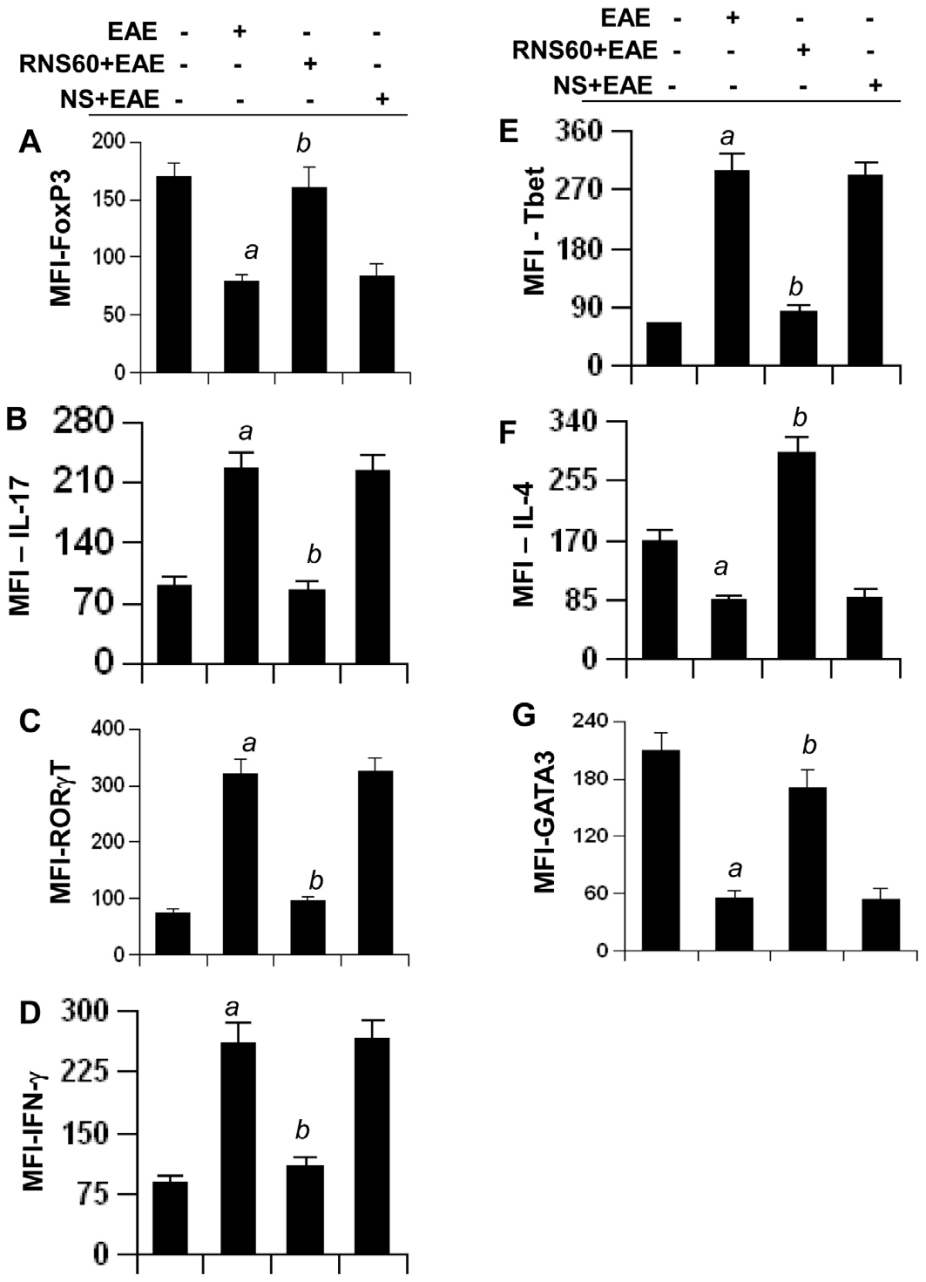
Regulation of Tregs, Th1, Th2, and Th17 cells in EAE mice by RNS60 treatment. EAE mice were treated with RNS60 or NS (300 µl/mouse/d via i.p. injection) from the onset of acute phase (8 dpt). On 16 dpt (peak of acute phase), spleens were harvested and splenocytes were stimulated with MBP in the absence of RNS60 or NS followed by analysis of Foxp3 (A), IFN-γ (B), T-bet (C), IL-17 (D), RORγt (E), IL-4 (F), and GATA3 (G) in CD4+ T cells by FACS. The MFI of Foxp3, IFN-γ, T-bet, IL-17, RORγt, IL-4, and GATA3 in CD4+ population was calculated by using CellQuest software. Data are mean ± S.D. of three different experiments. *^a^p<0.001* vs control; *^b^p<0.001* vs MBP.

Since RNS60 protected Tregs, suppressed Th17 and switched Th1 to Th2 in MBP-primed T cells, we investigated whether RNS60 treatment was capable of executing such immunomodulatory effect *in vivo* in EAE mice. EAE mice receiving RNS60 and NS from 8 dpt were sacrificed on 16 dpt, followed by flow cytometric analysis of splenocytes for Foxp3 (Tregs), IL-17 & RORγt (Th17), IFN-γ & T-bet (Th1), and IL-4 & GATA3 (Th2). As expected, induction of EAE markedly decreased CD4+Foxp3+ Tregs (Fig. 11A), increased the levels of CD4+IL-17+ (Fig. 11B) and CD4+RORγt+ (Fig. 11C) Th17 cells, upregulated the levels of CD4+IFN-γ+ (Fig. 11D) and CD4+T-bet+ (Fig. 11E) Th1 cells, and down-regulated CD4+IL-4+ (Fig. 11F) and CD4+GATA3+ (Fig. 11G) Th2 cells. However, treatment of EAE mice with RNS60, but not NS, led to the protection of CD4+Foxp3+ Tregs (Fig. 11A), suppression of CD4+IL-17+ (Fig. 11B) and CD4+RORγt+ (Fig. 11C) Th17 cells, decrease in CD4+IFN-γ+ (Fig. 11D) and CD4+T-bet+ (Fig. 11E) Th1 cells, and increase in CD4+IL-4+ (Fig. 11F) and CD4+GATA3+ (Fig. 11G) Th2 cells, compared to untreated EAE mice.

### RNS60 suppresses EAE in mice via Tregs

**Figure 12:**
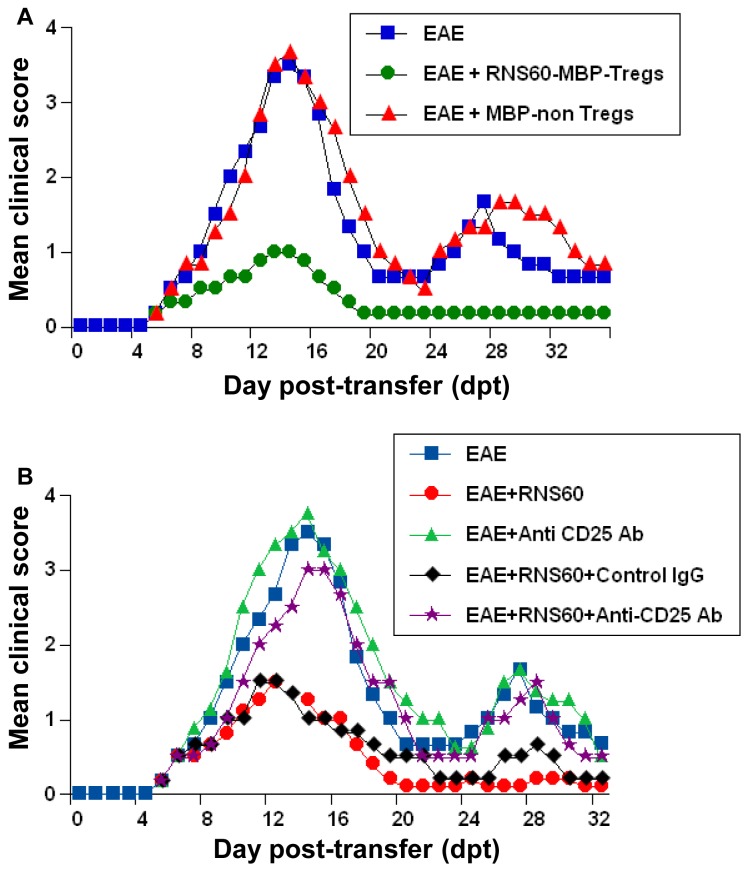
RNS60 protects mice from EAE via Tregs. A) MBP-primed T cells isolated from MBP-immunized donor mice were re-primed with MBP in the presence of 10% v/v RNS60 for 2 d followed by purification of CD4+CD25+ Tregs. In a parallel experiment, EAE was induced in female SJL/J mice by adoptive transfer of MBP-primed T cells and from 4 dpt, mice were treated (i.p.) once with RNS60-induced MBP-primed Tregs (1×10^6^ Tregs/mouse). Purified CD4+CD25− MBP-primed non-Tregs were also used for comparison. Five mice were included in each group. Mice (n = 5) were examined for clinical symptoms daily until 36 dpt. B) EAE was induced in female SJL/J mice by adoptive transfer of MBP-primed T cells and from 1 dpt, mice were treated with RNS60 (dpt 1–8, alternate day; dpt 9–16, daily; dpt 17 onwards, alternate day) followed by one i.p. injection of anti-CD25 antibody (50 µg/mouse) on 2 dpt. One group of mice also received same amount of control IgG. Mice (n = 5) were examined for clinical symptoms daily until 33 dpt.

Next, in order to test the functional significance of RNS60-mediated increase in Treg activity, we examined whether RNS60 protected mice from clinical symptoms of EAE via Tregs. At first, we checked whether RNS60-induced MBP-primed Tregs were capable of suppressing the adoptive transfer of EAE in female SJL/J mice. A single injection of RNS60-induced Tregs on 4 dpt markedly suppressed the clinical symptoms of EAE in recipient mice in acute as well as relapsing phases of the disease (Fig. 12A). This result was specific, as CD4+CD25− non-Tregs remained unable to inhibit the disease process of RR-EAE (Fig. 12A). These results suggest RNS60-induced Tregs are capable of suppressing EAE.

Next, we examined whether RNS60 treatment also protected EAE via Tregs. Therefore, during RNS60 treatment, the function of Tregs was blocked *in vivo* in EAE mice by anti-CD25 antibody. As evident from Figure 12B, RNS60 treatment ameliorated clinical symptoms of RR-EAE. However, functional blocking anti-CD25 antibody almost completely abrogated the RNS60-mediated protective effect on EAE mice (Fig. 12B). This result was specific as control IgG had no such effect (Fig. 12B). Together, these results delineate an important role of Tregs in RNS60-mediated protection of EAE.

## Discussion

MS is an autoimmune disease resulting from activation and proliferation of myelin-reactive T cells that cross the blood-brain barrier to enter the CNS. Once there, these cells initiate, promote, and aggravate multifaceted inflammatory and degenerative insults ultimately leading to demyelination and axonal injury. There are many hypotheses to explain how auto-reactive T cells might be activated, including microbial, viral infection, and molecular mimicry [30], [32], [33]. However, no consensus has been achieved due to the lack of convincing evidence. Major advancement occurred after the concept of regulatory T cells (Tregs) became clear. Although several different types of Tregs with specific surface or secretory molecules have been identified, Foxp3 is generally considered as the signature molecule that is associated with Treg properties [1], [4]. The primary role of Tregs in the immune system is to suppress unwanted activation of responder cells including Th1 and Th17 and to maintain immune homeostasis [1], [2], [4]. Therefore, it is likely that dysfunction of Tregs could be a major cause for the activation of myelin-reactive T cells in MS.

It has been shown that there is a significant decrease in the number of CD4^+^Foxp3^+^ T cells as well as the expression level of Foxp3 in relapsing-remitting MS and other lymphoproliferative autoimmune disorders [7], [9], [34]. Hence, the upregulation of Foxp3+ Tregs might be useful for suppressing the activation of autoimmune Th1 and Th17 cells and controlling autoimmune disorders. Accordingly, the identification of drugs and associated mechanisms that could upregulate Foxp3 is an important area of study. Although other drugs and approaches exist to execute immunomodulation, here we introduce a simple saline-based agent to achieve immunomodulation. Upon subjecting normal saline to Taylor-Couette-Poiseuille (TCP) turbulence in the presence of elevated oxygen pressure, Revalesio Corporation (Tacoma, WA) has generated RNS60, which does not contain any active pharmaceutical ingredient [12]. Due to TCP turbulence, RNS60 is proposed to contain charge-stabilized nanostructures consisting of an oxygen nanobubble core surrounded by an electrical double-layer at the liquid/gas interface. Nanobubbles have been suggested for potential use in biomedical imaging and drug delivery [35], [36], but have not demonstrated to have direct biological effects.

Here we demonstrate the first evidence that saline generated due to TCP turbulence is capable of enriching Foxp3^+^ Tregs. Our conclusion is based on the following observations. *First,* as we reported earlier, MBP-priming reduced the expression of Foxp3 in T cells. However, RNS60 markedly inhibited the loss of Foxp3 in MBP-primed T cells. This inhibition was also specific as other saline preparations like NS (normal saline of the same batch), RNS10.3 (TCP-modified saline without excess oxygen) and PNS60 (saline containing oxygen in the absence of TCP modification) had no effect on the loss of Foxp3. *Second,* Foxp3+ regulatory T cells are also characterized by CD25, CD62L, CTLA4, CD73 etc. Accordingly, we have found loss of CD25, CD62L, CTLA4, CD73 in MBP-primed LNC compared to normal LNC. Again RNS60 treatment inhibited the loss of CD25, CD62L, CTLA4 and CD73 in MBP-primed LNC. These results were specific as neither MBP-priming nor RNS60 treatment had any effect on CD4. The unperturbed expression of CD4 probably implies that either suppression of Foxp3 by MBP-priming or upregulation of Foxp3 by RNS60 treatment is not due to any reduction of CD4^+^ cells. Finally, Tregs are also known as suppressor T cells as they suppress immune responses of other cells. Accordingly, suppression of IFN-γ expression in MOG-primed tomato red T cells by RNS60-induced Tregs suggests that RNS60-induced Tregs are functionally active.

Mechanisms by which Tregs could be restored during an autoimmune insult are poorly understood. Recently, we have delineated that NO is a critical regulator of Foxp3 and Tregs [17]. While blocking NO either by inhibiting iNOS or direct scavenging of NO or by pharmacological drugs restores the expression of Foxp3 in MBP-primed T cells, NO donors decrease Foxp3 [17]. Therefore, we tested the hypothesis that RNS60 might enrich Tregs via regulating NO production. Our results that RNS60 inhibits the production of NO and the expression of iNOS in MBP-primed LNC and splenocytes and that a NO donor abrogates RNS60-mediated restoration and/or upregulation of Foxp3 suggest that RNS60 boosts Tregs via suppression of NO production.

The major function of Tregs is to maintain immune homeostasis. While Tregs suppress the proliferation of autoimmune Th1 cells by secreting TGF-β and IL-10, Tregs release IL-35 to control the proliferation of autoimmune Th17 cells [1], [4], [34], [37], [38]. Accordingly, we found that RNS60 suppresses both Th1 and Th17 immune responses. Theoretically, Tregs should also suppress Th2 via releasing TGF-β and IL-35 [4], [38], [39]. However, in our study, RNS60 suppressed the Th1 and Th17 responses, while stimulating the Th2 response. It is also possible as Tregs may contribute to Th2 polarization. For example, McKee and Pearce [22] have demonstrated that Tregs contribute to Th2 polarization during Helminth infection by suppressing the development of Th1 response. Similarly, Kohm et al [21] have shown that supplementation of Tregs by adoptive transfer before active and adoptive EAE induction significantly reduces the severity of clinical disease, potentially by promoting a disease protective Th2 immune response. Here we also must remember that Tregs produce substantial amount of IL-10, a cytokine that is also produced by Th2 cells. Therefore, whether the stimulation of Th2 response by RNS60 is a direct effect of RNS60, an indirect effect via enrichment of Tregs, or both, needs further study.

Because Tregs have been implicated in the pathogenesis of autoimmune diseases, we examined the effect of RNS60 treatment on the disease process of RR-EAE. Here we delineate the first evidence that TCP-modified saline RNS60 inhibits the disease process of RR-EAE. Adoptively-transferred MBP-primed T cells remained unable to induce clinical symptoms of EAE in female SJL/J mice receiving RNS60. In contrast, MBP-primed T cells induced EAE in mice receiving NS. From a therapeutic point of view, it is important to test whether a drug candidate is efficacious when administered after the onset of disease symptoms. RNS60 fulfilled this requirement and inhibited the progression of RR-EAE when administered either early or at a late stage of the disease progression. When we examined the effect of RNS60 on the encephalitogenicity of T cells, we found that adoptive transfer of MBP-primed T cells and NS-treated MBP-primed T cells but not that of RNS60-treated MBP-primed T cells induced the clinical symptoms of EAE in naïve female SJL/J mice. Therapeutic treatment of EAE animals with RNS60 was also capable of inhibiting the invasion of mononuclear cells into cerebellum and spinal cord, as well as the expression of inflammatory molecules (iNOS and IL-1β), and restored myelination and the expression of myelin genes within the CNS. In order to directly prove the involvement of Tregs, we transferred RNS60-induced Tregs to EAE mice and, in a different experiment, used an anti-CD25 antibody in RNS60-treated EAE mice. Suppression of EAE by RNS60-induced Tregs and abrogation of RNS60-mediated protection of EAE by anti-CD25 antibody clearly suggest that RNS60-induced Tregs are capable of ameliorating EAE and that the effect of RNS60 treatment is Treg-dependent.

Tysabri and different forms of interferon-γ (IFN-γ) are currently used to treat MS. However, reduced effectiveness and severe toxic effects over chronic use, as well as treatment costs, often limit these available therapies. For example, IFN-γ has a number of side effects including flu-like symptoms, menstrual disorders in women, decrease in neutrophil and white blood cell count, increase in AST and ALT levels, and development of neutralizing antibodies to IFN-γ [10], [40], [41]. Similarly, treatment with Tysabri can cause lung infection, breathing problems, chest pain, wheezing, urinary tract infection, vaginitis, nausea, vomiting, and liver damage. Tysabri also increases the chance of getting a severe brain infection leading to progressive multifocal encephalopathy, which may cause disability and death. RNS60 offers several potential advantages over existing therapies. It has a simple chemical composition (NaCl, oxygen, and water) and does not contain any active pharmaceutical ingredients. Instead, its biological activity is achieved by physical processing. Because of its unique nature, RNS60 has not shown any cytotoxic effects and hence may be expected to have little or no side effects compared to existing immune-modulatory drugs for MS.

In summary, we have demonstrated that RNS60 upregulates anti-autoimmune Treg/Th2 cells, down-regulates autoimmune Th17/Th1 cells and blocks the disease process of RR- EAE when administered either prophylactially or therapeutcially. These results highlight a novel immunomodulatory role of RNS60 and suggest that this simple modified saline may be explored for therapeutic intervention in MS.

## Supporting Information

Figure S1 **Mass spectrometric analyses of NS, PNS60 and RNS60.** To examine compositional differences in NS, PNS60 and RNS60, the LC-Q-TOF system was configured with an electrospray ionization interface (ESI) and the analysis was performed in both positive and negative modes. To facilitate visual comparison, the 100 to 1000 *m/z* scan range for each sample was separated into 9 segments of 100 *m/z* each and printed as part of the study data. The extracted segments from each of PNS60 and RNS60 were compared to the corresponding extracted segments for NS. Only a part of it is shown (A, solvent; B, NS; C, PNS60; D, RNS60).
(TIF)

Figure S2 **Effect of RNS60 on the expression of iNOS and Foxp3 in MBP-primed splenocytes.** Splenocytes isolated from MBP-immunized donor mice were stimulated with MBP for 24 h in the presence or absence of 10% (v/v) RNS60 or NS followed by monitoring the level of nitrite (A) and the expression of iNOS and Foxp3 mRNAs by semi-quantitative RT-PCR (B). The mRNA expression of iNOS (C) and Foxp3 (D) was also monitored by real-time PCR. Results are mean ± SD of three different experiments. *^a^p*<0.0001 versus control; *^b^p*<0.0001 versus MBP.
(TIF)

Figure S3 **Suppression of Th1 cells by RNS60, but not NS, PNS60 and RNS10.3.** LNC isolated from MBP-immunized mice were stimulated with MBP in the presence of 10% v/v of RNS60, NS, PNS60, or RNS10.3. A) After 72 h of stimulation, T cells were incubated with appropriately diluted PE-conjugated anti-IFN-γ and FITC-conjugated anti-CD4 Abs followed by FACS analysis. The percentage of relevant cells is indicated in their respective quadrants. B) The mean fluorescence intensity (MFI) of IFN-γ in CD4+ population was calculated by using CellQuest software. C) Supernatants were assayed for IFN-γ by ELISA. Data are mean ± SD of three different experiments. *^a^p<0.001* vs control; *^b^p<0.001* vs MBP. D) LNC isolated from MBP-immunized donor mice were stimulated with MBP in the presence of 10% v/v of RNS60 and NS followed by FACS analysis using appropriately-diluted PE-conjugated anti-T-bet and FITC-conjugated anti-CD4 Abs. E) The MFI of T-bet in CD4+ population was calculated by using CellQuest software. Data are mean ± SD of three different experiments. *^a^p<0.001* vs control; *^b^p<0.001* vs MBP. F) LNC isolated from MBP-immunized donor mice were stimulated with MBP in the presence or absence of RNS60 and NS, respectively, for 48 h followed by monitoring the mRNA expression of IFN-γ and T-bet by semi-quantitative RT-PCR. Results represent three independent experiments.
(TIF)

## References

[1] Coffer PJ, Burgering BM (2004) Forkhead-box transcription factors and their role in the immune system. Nat Rev Immunol 4: 889–899. 15516968 10.1038/nri1488

[2] Hori S, Nomura T, Sakaguchi S (2003) Control of regulatory T cell development by the transcription factor Foxp3. Science 299: 1057–1061. 12522256 10.1126/science.1079490

[3] Sakaguchi S, Powrie F (2007) Emerging challenges in regulatory T cell function and biology. Science 317: 627–629. 17673654 10.1126/science.1142331

[4] Sakaguchi S (2005) Naturally arising Foxp3-expressing CD25+CD4+ regulatory T cells in immunological tolerance to self and non-self. Nat Immunol 6: 345–352. 15785760 10.1038/ni1178

[5] Huan J, Culbertson N, Spencer L, Bartholomew R, Burrows GG, et al (2005) Decreased FOXP3 levels in multiple sclerosis patients. J Neurosci Res 81: 45–52. 15952173 10.1002/jnr.20522

[6] Viglietta V, Baecher-Allan C, Weiner HL, Hafler DA (2004) Loss of functional suppression by CD4+CD25+ regulatory T cells in patients with multiple sclerosis. J Exp Med 199: 971–979. 15067033 10.1084/jem.20031579 PMC2211881

[7] Paust S, Cantor H (2005) Regulatory T cells and autoimmune disease. Immunol Rev 204: 195–207. 15790360 10.1111/j.0105-2896.2005.00247.x

[8] McGeachy MJ, Stephens LA, Anderton SM (2005) Natural recovery and protection from autoimmune encephalomyelitis: contribution of CD4+CD25+ regulatory cells within the central nervous system. J Immunol 175: 3025–3032. 16116190 10.4049/jimmunol.175.5.3025

[9] Venken K, Hellings N, Thewissen M, Somers V, Hensen K, et al (2008) Compromised CD4+ CD25(high) regulatory T-cell function in patients with relapsing-remitting multiple sclerosis is correlated with a reduced frequency of FOXP3-positive cells and reduced FOXP3 expression at the single-cell level. Immunology 123: 79–89. 17897326 10.1111/j.1365-2567.2007.02690.x PMC2433271

[10] Pahan K (2010) Neuroimmune pharmacological control of EAE. J Neuroimmune Pharmacol 5: 165–167. 20414732 10.1007/s11481-010-9219-6 PMC2881219

[11] Pahan K (2011) Immunomodulation of experimental allergic encephalomyelitis by cinnamon metabolite sodium benzoate. Immunopharmacol Immunotoxicol 33: 586–593. 21425926 10.3109/08923973.2011.561861 PMC3206174

[12] Khasnavis S, Jana A, Roy A, Mazumder M, Bhushan B, et al (2012) Suppression of Nuclear Factor-kappaB Activation and Inflammation in Microglia by Physically Modified Saline. J Biol Chem 287: 29529–29542. 22753407 10.1074/jbc.M111.338012 PMC3436140

[13] Dasgupta S, Jana M, Zhou Y, Fung YK, Ghosh S, et al (2004) Antineuroinflammatory effect of NF-kappaB essential modifier-binding domain peptides in the adoptive transfer model of experimental allergic encephalomyelitis. J Immunol 173: 1344–1354. 15240729 10.4049/jimmunol.173.2.1344

[14] Dasgupta S, Zhou Y, Jana M, Banik NL, Pahan K (2003) Sodium phenylacetate inhibits adoptive transfer of experimental allergic encephalomyelitis in SJL/J mice at multiple steps. J Immunol 170: 3874–3882. 12646656 10.4049/jimmunol.170.7.3874

[15] Brahmachari S, Pahan K (2007) Sodium benzoate, a food additive and a metabolite of cinnamon, modifies T cells at multiple steps and inhibits adoptive transfer of experimental allergic encephalomyelitis. J Immunol 179: 275–283. 17579047 10.4049/jimmunol.179.1.275 PMC1976122

[16] Mondal S, Roy A, Pahan K (2009) Functional blocking monoclonal antibodies against IL-12p40 homodimer inhibit adoptive transfer of experimental allergic encephalomyelitis. J Immunol 182: 5013–5023. 19342681 10.4049/jimmunol.0801734 PMC2721330

[17] Brahmachari S, Pahan K (2010) Myelin basic protein priming reduces the expression of Foxp3 in T cells via nitric oxide. J Immunol 184: 1799–1809. 20083653 10.4049/jimmunol.0804394 PMC2855656

[18] Brahmachari S, Pahan K (2009) Suppression of regulatory T cells by IL-12p40 homodimer via nitric oxide. J Immunol 183: 2045–2058. 19587012 10.4049/jimmunol.0800276 PMC2713791

[19] Pahan K, Sheikh FG, Namboodiri AM, Singh I (1997) Lovastatin and phenylacetate inhibit the induction of nitric oxide synthase and cytokines in rat primary astrocytes, microglia, and macrophages. J Clin Invest 100: 2671–2679. 9389730 10.1172/JCI119812 PMC508470

[20] Dasgupta S, Jana M, Liu X, Pahan K (2003) Role of very-late antigen-4 (VLA-4) in myelin basic protein-primed T cell contact-induced expression of proinflammatory cytokines in microglial cells. J Biol Chem 278: 22424–22431. 12690109 10.1074/jbc.M301789200 PMC1955481

[21] Kohm AP, Carpentier PA, Anger HA, Miller SD (2002) Cutting edge: CD4+CD25+ regulatory T cells suppress antigen-specific autoreactive immune responses and central nervous system inflammation during active experimental autoimmune encephalomyelitis. J Immunol 169: 4712–4716. 12391178 10.4049/jimmunol.169.9.4712

[22] McKee AS, Pearce EJ (2004) CD25+CD4+ cells contribute to Th2 polarization during helminth infection by suppressing Th1 response development. J Immunol 173: 1224–1231. 15240714 10.4049/jimmunol.173.2.1224

[23] El-behi M, Rostami A, Ciric B (2010) Current views on the roles of Th1 and Th17 cells in experimental autoimmune encephalomyelitis. J Neuroimmune Pharmacol 5: 189–197. 20107924 10.1007/s11481-009-9188-9 PMC2866798

[24] Chaudhry A, Rudra D, Treuting P, Samstein RM, Liang Y, et al (2009) CD4+ regulatory T cells control TH17 responses in a Stat3-dependent manner. Science 326: 986–991. 19797626 10.1126/science.1172702 PMC4408196

[25] Shi G, Cox CA, Vistica BP, Tan C, Wawrousek EF, et al (2008) Phenotype switching by inflammation-inducing polarized Th17 cells, but not by Th1 cells. J Immunol 181: 7205–7213. 18981142 10.4049/jimmunol.181.10.7205 PMC2665021

[26] Schrempf W, Ziemssen T (2007) Glatiramer acetate: mechanisms of action in multiple sclerosis. Autoimmun Rev 6: 469–475. 17643935 10.1016/j.autrev.2007.02.003

[27] Zhu J, Paul WE (2010) CD4+ T cell plasticity-Th2 cells join the crowd. Immunity 32: 11–13. 20152167 10.1016/j.immuni.2010.01.001 PMC3494417

[28] Dasgupta S, Roy A, Jana M, Hartley DM, Pahan K (2007) Gemfibrozil ameliorates relapsing-remitting experimental autoimmune encephalomyelitis independent of peroxisome proliferator-activated receptor-alpha. Mol Pharmacol 72: 934–946. 17625103 10.1124/mol.106.033787

[29] Kuerten S, Lehmann PV (2011) The immune pathogenesis of experimental autoimmune encephalomyelitis: lessons learned for multiple sclerosis? J Interferon Cytokine Res 31: 907–916. 21936633 10.1089/jir.2011.0072

[30] Martin R, McFarland HF, McFarlin DE (1992) Immunological aspects of demyelinating diseases. Annu Rev Immunol 10: 153–187. 1375472 10.1146/annurev.iy.10.040192.001101

[31] Chun J, Hartung HP (2010) Mechanism of action of oral fingolimod (FTY720) in multiple sclerosis. Clin Neuropharmacol 33: 91–101. 20061941 10.1097/WNF.0b013e3181cbf825 PMC2859693

[32] Sabatino JJ Jr, Rosenthal KM, Evavold BD (2010) Manipulating antigenic ligand strength to selectively target myelin-reactive CD4+ T cells in EAE. J Neuroimmune Pharmacol 5: 176–188. 19904613 10.1007/s11481-009-9181-3 PMC2866818

[33] Sinha S, Subramanian S, Emerson-Webber A, Lindner M, Burrows GG, et al (2010) Recombinant TCR ligand reverses clinical signs and CNS damage of EAE induced by recombinant human MOG. J Neuroimmune Pharmacol 5: 231–239. 19789980 10.1007/s11481-009-9175-1 PMC2866769

[34] Ziegler SF (2006) FOXP3: of mice and men. Annu Rev Immunol 24: 209–226. 16551248 10.1146/annurev.immunol.24.021605.090547

[35] Bhaskar S, Tian F, Stoeger T, Kreyling W, de la Fuente JM, et al (2010) Multifunctional Nanocarriers for diagnostics, drug delivery and targeted treatment across blood-brain barrier: perspectives on tracking and neuroimaging. Part Fibre Toxicol 7: 3. 20199661 10.1186/1743-8977-7-3 PMC2847536

[36] Rapoport N, Gao Z, Kennedy A (2007) Multifunctional nanoparticles for combining ultrasonic tumor imaging and targeted chemotherapy. J Natl Cancer Inst 99: 1095. 17623798 10.1093/jnci/djm043

[37] Niedbala W, Wei XQ, Cai B, Hueber AJ, Leung BP, et al (2007) IL-35 is a novel cytokine with therapeutic effects against collagen-induced arthritis through the expansion of regulatory T cells and suppression of Th17 cells. Eur J Immunol 37: 3021–3029. 17874423 10.1002/eji.200737810

[38] Xu D, Liu H, Komai-Koma M, Campbell C, McSharry C, et al (2003) CD4+CD25+ regulatory T cells suppress differentiation and functions of Th1 and Th2 cells, Leishmania major infection, and colitis in mice. J Immunol 170: 394–399. 12496424 10.4049/jimmunol.170.1.394

[39] Reddy J, Waldner H, Zhang X, Illes Z, Wucherpfennig KW, et al (2005) Cutting edge: CD4+CD25+ regulatory T cells contribute to gender differences in susceptibility to experimental autoimmune encephalomyelitis. J Immunol 175: 5591–5595. 16237044 10.4049/jimmunol.175.9.5591

[40] Miller A (1997) Current and investigational therapies used to alter the course of disease in multiple sclerosis. South Med J 90: 367–375. 9114824 10.1097/00007611-199704000-00001

[41] Cohen JA, Reingold SC, Polman CH, Wolinsky JS (2012) Disability outcome measures in multiple sclerosis clinical trials: current status and future prospects. Lancet Neurol 11: 467–476. 22516081 10.1016/S1474-4422(12)70059-5

