# Supplementary figures and images for: Protection of Tregs, Suppression of Th1 and Th17 Cells, and Amelioration of Experimental Allergic Encephalomyelitis by a Physically-Modified Saline
Source: PLoS One. 2012 Dec 20;7(12):e51869. doi: 10.1371/journal.pone.0051869 (PMC3527485; doi:10.1371/journal.pone.0051869)

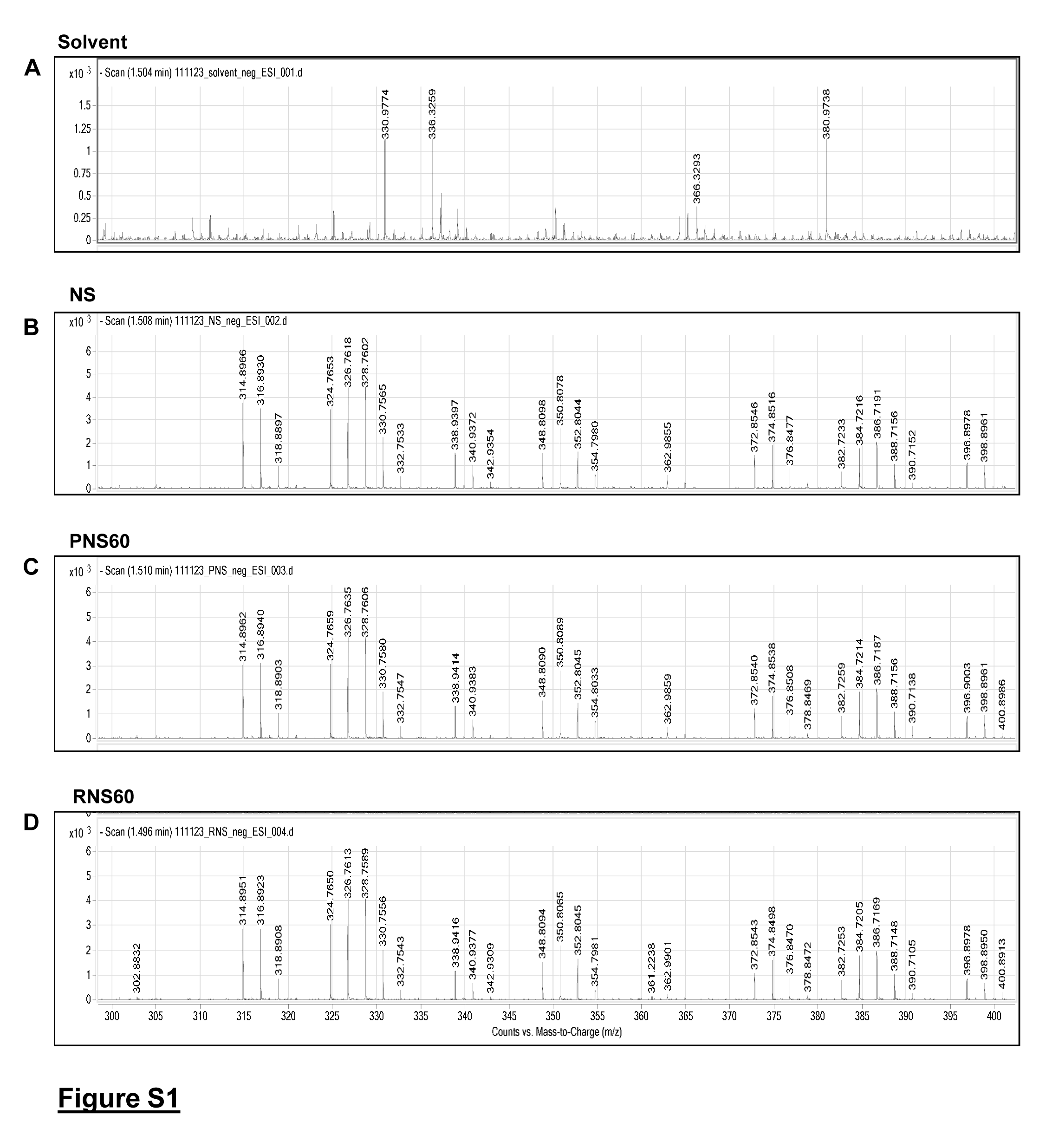

Supplement: Figure S1 — Mass spectrometric analyses of NS, PNS60 and RNS60. To examine compositional differences in NS, PNS60 and RNS60, the LC-Q-TOF system was configured with an electrospray ionization interface (ESI) and the analysis was performed in both positive and negative modes. To facilitate visual comparison, the 100 to 1000 m/z scan range for each sample was separated into 9 segments of 100 m/z each and printed as part of the study data. The extracted segments from each of PNS60 and RNS60 were compared to the corresponding extracted segments for NS. Only a part of it is shown (A, solvent; B, NS; C, PNS60; D, RNS60). (TIF) [file pone.0051869.s001.tif]

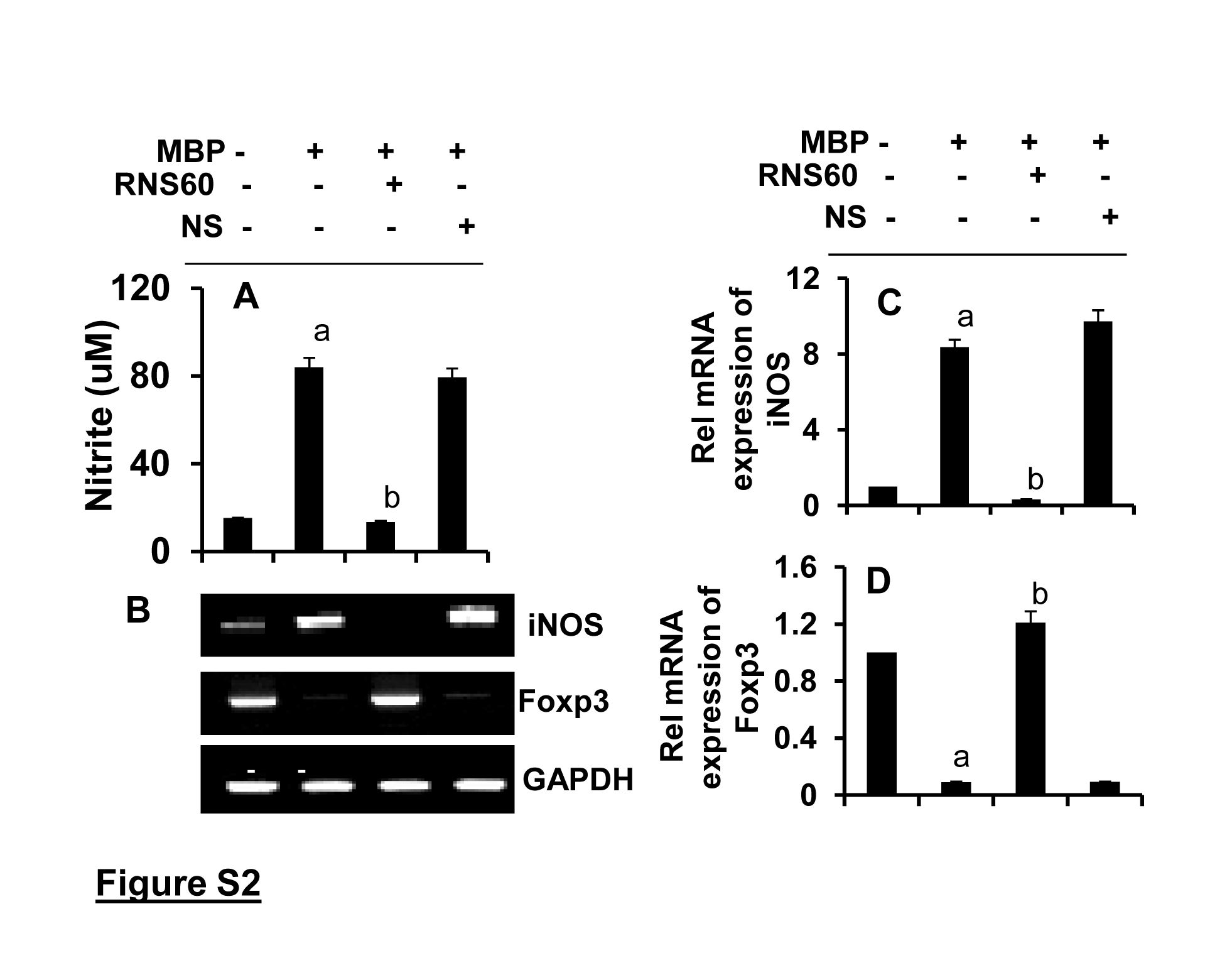

Supplement: Figure S2 — Effect of RNS60 on the expression of iNOS and Foxp3 in MBP-primed splenocytes. Splenocytes isolated from MBP-immunized donor mice were stimulated with MBP for 24 h in the presence or absence of 10% (v/v) RNS60 or NS followed by monitoring the level of nitrite (A) and the expression of iNOS and Foxp3 mRNAs by semi-quantitative RT-PCR (B). The mRNA expression of iNOS (C) and Foxp3 (D) was also monitored by real-time PCR. Results are mean ± SD of three different experiments. ap<0.0001 versus control; bp<0.0001 versus MBP. (TIF) [file pone.0051869.s002.tif]

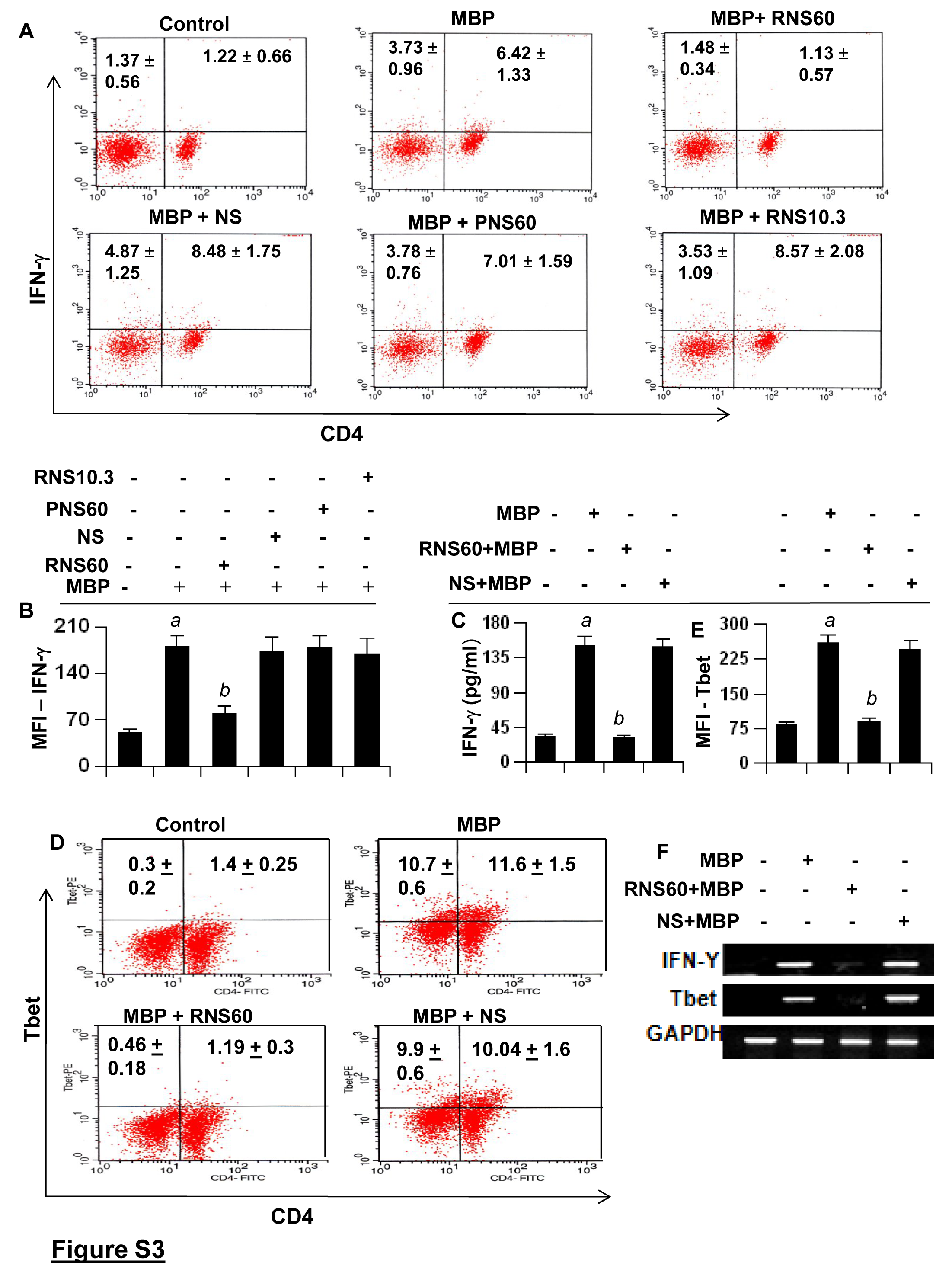

Supplement: Figure S3 — Suppression of Th1 cells by RNS60, but not NS, PNS60 and RNS10.3. LNC isolated from MBP-immunized mice were stimulated with MBP in the presence of 10% v/v of RNS60, NS, PNS60, or RNS10.3. A) After 72 h of stimulation, T cells were incubated with appropriately diluted PE-conjugated anti-IFN-γ and FITC-conjugated anti-CD4 Abs followed by FACS analysis. The percentage of relevant cells is indicated in their respective quadrants. B) The mean fluorescence intensity (MFI) of IFN-γ in CD4+ population was calculated by using CellQuest software. C) Supernatants were assayed for IFN-γ by ELISA. Data are mean ± SD of three different experiments. ap<0.001 vs control; bp<0.001 vs MBP. D) LNC isolated from MBP-immunized donor mice were stimulated with MBP in the presence of 10% v/v of RNS60 and NS followed by FACS analysis using appropriately-diluted PE-conjugated anti-T-bet and FITC-conjugated anti-CD4 Abs. E) The MFI of T-bet in CD4+ population was calculated by using CellQuest software. Data are mean ± SD of three different experiments. ap<0.001 vs control; bp<0.001 vs MBP. F) LNC isolated from MBP-immunized donor mice were stimulated with MBP in the presence or absence of RNS60 and NS, respectively, for 48 h followed by monitoring the mRNA expression of IFN-γ and T-bet by semi-quantitative RT-PCR. Results represent three independent experiments. (TIF) [file pone.0051869.s003.tif]
